# Bahadur representations of M-estimators and their applications in general linear models

**DOI:** 10.1186/s13660-018-1715-x

**Published:** 2018-05-22

**Authors:** Hongchang Hu

**Affiliations:** School of Mathematics and Statistics, Hubei Normal University, Huangshi, China

**Keywords:** 62J05, 62F35, 62M10, Linear regression models, M-estimate, Bahadur representation, Normal distribution, Rate of strong convergence

## Abstract

Consider the linear regression model

$$y_{i}=x_{i}^{T}\beta+e_{i},\quad i=1,2, \ldots,n, $$

where $e_{i}=g(\ldots,\varepsilon_{i-1},\varepsilon_{i})$ are general dependence errors. The Bahadur representations of M-estimators of the parameter *β* are given, by which asymptotically the theory of M-estimation in linear regression models is unified. As applications, the normal distributions and the rates of strong convergence are investigated, while $\{\varepsilon_{i},i\in{Z}\}$ are *m*-dependent, and the martingale difference and $(\varepsilon,\psi)$-weakly dependent.

## Introduction

Consider the following linear regression model:

1.1 $$ y_{i}=x_{i}^{T}\beta+e_{i},\quad i=1,2, \ldots,n, $$

where $\beta=(\beta_{1},\ldots,\beta_{p})^{T}\in{R^{p}}$ is an unknown parametric vector, $x_{i}^{T}$ denotes the *i*th row of an $n\times{p}$ design matrix *X*, and $\{e_{i}\}$ are stationary dependence errors with a common distribution.

An M-estimate of *β* is defined as any value of *β* minimizing

1.2 $$ \sum_{i=1}^{n}\rho\bigl(y_{i}-x_{i}^{T} \beta\bigr) $$

for a suitable choice of the function *ρ*, or any solution for *β* of the estimating equation

1.3 $$ \sum_{i=1}^{n}\psi\bigl(y_{i}-x_{i}^{T} \beta\bigr)x_{i}=0 $$

for a suitable choice of *ψ*.

There is a body of statistical literature dealing with linear regression models with independent and identically distributed (i.i.d.) random errors, see e.g. Babu [1], Bai et al. [2], Chen [7], Chen and Zhao [8], He and Shao [24], Gervini and Yohai [23], Huber and Ronchetti [28], Xiong and Joseph [50], Salibian-Barrera et al. [44]. Recently, linear regression models with serially correlated errors have attracted increasing attention from statisticians; see, for example, Li [33], Wu [49], Maller [38], Pere [41], Hu [25, 26]. Over the last 40 years, M-estimators in linear regression models have been investigated by many authors. Let $\{\eta_{i}\}$ be i.i.d. random variables. Koul [30] discussed the asymptotic behavior of a class of M-estimators in the model (1.1) with long range dependence errors $e_{i}=G(\eta_{i})$. Wu [49] and Zhou and Shao [52] discussed the model (1.1) with $e_{i}=G(\ldots,\eta_{i-1},\eta_{i})$ and derived strong Bahadur representations of M-estimators and a central limit theorem. Zhou and Wu [53] considered the model (1.1) with $e_{i}=\sum_{j=0}^{\infty}{a}_{j}\eta_{i-j}$, and obtained some asymptotic results including consistency of robust estimates. Fan et al. [20] investigated the model (1.1) with the errors $e_{i}=f(e_{i-1})+\eta_{i}$ and established the moderate deviations and strong Bahadur representations for M-estimators. Wu [47] discussed strong consistency of an M-estimator in the model (1.1) for negatively associated samples. Fan [19] considered the model (1.1) with *φ*-mixing errors, and the moderate deviations for the M-estimators. In addition, Berlinet et al. [4], Boente and Fraiman [5], Chen et al. [6], Cheng et al. [9], Gannaz [22], Lô and Ronchetti [37], Valdora and Yohai [45] and Yang [51] have also studied some asymptotic properties of M-estimators in nonlinear models. However, no people have investigated a unified the theory of M-estimation in linear regression models with more general errors.

In this paper, we assume that

1.4 $$ e_{i}=g(\ldots,\varepsilon_{i-1},\varepsilon_{i}), $$

where $g(\cdot)$ is a measurable function such that $e_{i}$ is a proper random variable, and $\{\varepsilon_{i},i\in{Z}\}$ (where *Z* is the set of integers) are very general random variables, including *m*-dependent, martingale difference, $(\varepsilon,\psi)$-weakly dependent, and so on.

We try to investigate the unified the theory of M-estimation in the linear regression model. In the article, we use the idea of Wu [49] to study the Bahadur representative of M-estimator, and we extend some results to general errors. The paper is organized as follows. In Sect. 2, the weak and strong linear representation of an M-estimate of the vector regression parameter *β* in the model (1.1) are presented. Section 3 contains some applications of our results, including the *m*-dependent, $(\varepsilon,\psi)$-weakly dependent, martingale difference. In Sect. 4, proofs of the main results are given.

## Main results

In the section, we investigate the weak and strong linear representation of an M-estimate of the vector regression parameter *β* in the model (1.1). Without loss of generality, we assume that the true parameter $\beta=0$. We start with some notation and assumptions.

For a vector $v=(v_{1},\ldots,v_{p})$, let $|v|=(\sum_{i=1}^{p}{v_{i}}^{2})^{\frac{1}{2}}$. A random vector *V* is said to be in $L^{q},q>0$, if $E(|V|^{q})<\infty$. Let $\Vert V \Vert _{q}=E(|V|^{q})^{\frac{1}{q}}$, $\Vert V \Vert = \Vert V \Vert _{2}$, $\Sigma_{n}=\sum_{i=1}^{n}{x_{i}}x_{i}^{T}=X^{T}X$ and assume that $\Sigma_{n}$ is positive definite for large enough *n*. Let $x_{in}=\Sigma_{n}^{-\frac{1}{2}}x_{i},\beta_{n}=\Sigma_{n}^{-\frac{1}{2}}\beta $. Then the model (1.1) can be written as

2.1 $$ y_{i}=x_{in}^{T}\beta_{n}+e_{i},\quad i=1,2, \ldots,n, $$

with $\sum_{i=1}^{n}{x_{in}}x_{in}^{T}=I_{p}$, where $I_{p}$ is an identity matrix of order *p*. Assume that *ρ* has derivative *ψ*. For $l\geq0$ and a function *f*, write $f\in{C^{l}}$ if *f* has derivatives up to *l*th order and $f^{(l)}$ is continuous. Define the function

2.2 $$ \psi_{k}(t;\mathcal{F}_{i})=E\bigl(\psi(e_{k}+t)| \mathcal{F}_{i}\bigr),\psi_{k}\bigl(t;\mathcal {F}_{i}^{*}\bigr)=E\bigl(\psi\bigl(e_{k}^{*}+t\bigr)| \mathcal{F}_{i}^{*}\bigr),\quad k\geq0, $$

where $\mathcal{F}_{i}^{*}=(\ldots,\varepsilon_{-1},\varepsilon '_{0},\varepsilon_{1},\ldots,\varepsilon_{i-1},\varepsilon_{i}), \mathcal{F}_{i}=(\ldots,\varepsilon_{-1},\varepsilon_{0},\varepsilon _{1},\ldots,\varepsilon_{i-1},\varepsilon_{i})$, let $\varepsilon'_{i}$ be an i.i.d. copy of $\varepsilon_{i}$, and $e_{k}^{*}=g(\mathcal{F}_{k}^{*})$.

Throughout the paper, we use the following assumptions.

1. $\rho(\cdot)$ is a convex function, $E\psi(e_{i})=0,0< E\psi^{2}(e_{i})$.
2. $\varphi(t)\equiv{E}\psi(e_{i}+t)$ has a strictly positive derivative at $t=0$.
3. $m(t)\equiv{E}|\psi(e_{i}+t)-\psi(e_{i})|^{2}$ is continuous at $t=0$.
4. $r_{n}\equiv\max_{1\leq{i}\leq{n}}|x_{in}|=\max_{1\leq {i}\leq{n}}(x_{i}^{T}\Sigma_{n}^{-1}x_{i})^{\frac{1}{2}}=o(1)$.
5. There exists a $\delta_{0}>0$ such that
  2.3 $$ L_{i}\equiv\sup_{ \vert s \vert , \vert t \vert \leq\delta_{0},s\neq{t}}\frac{ \vert \psi _{i+1}(s;\mathcal{F}_{i})-\psi_{i+1}(t;\mathcal{F}_{i}) \vert }{ \vert s-t \vert } \in{L^{1}}. $$
6. Let $\psi_{i}(\cdot;\mathcal{F}_{i-1})\in{C^{l}},l\geq0$. For some $\delta_{0}>0,\max_{1\leq{i}\leq{n}}\sup_{|\delta|\leq\delta _{0}} \Vert \psi_{i}^{(l)}(\delta;\mathcal{F}_{i-1}) \Vert <\infty$ and
  2.4 $$ \sum_{i=0}^{\infty}\sup_{ \vert \delta \vert < \delta_{0}} \bigl\Vert E\bigl(\psi _{i}^{(l)}(\delta; \mathcal{F}_{i-1})|\mathcal{F}_{0}\bigr)-E\bigl(\psi _{i}^{(l)}\bigl(\delta;\mathcal{F}_{i-1}^{*}\bigr)| \mathcal{F}_{0}^{*}\bigr) \bigr\Vert < \infty. $$
7. 2.5 $$\begin{aligned} &\sum_{i=0}^{\infty}\sup_{ \vert \delta \vert < \delta_{0}} \bigl\Vert E\bigl(\psi _{i}^{(l)}\bigl(\delta; \mathcal{F}_{i-1}^{*}\bigr)|\mathcal{F}_{0}\bigr)-E\bigl(\psi _{i}^{(l)}\bigl(\delta;\mathcal{F}_{i-1}^{*}\bigr)| \mathcal{F}_{-1}\bigr) \bigr\Vert < \infty, \end{aligned}$$
  2.6 $$\begin{aligned} &\sum_{i=0}^{\infty}\sup_{ \vert \delta \vert < \delta_{0}} \bigl\vert E\psi ^{(l)}(e_{i}+\delta)-E\psi^{(l)} \bigl(e_{i}^{*}+\delta\bigr) \bigr\vert < \infty. \end{aligned}$$

### Remark 1

Conditions (A1)–(A5) and (A6) are imposed in the M-estimation considering the theory of linear regression models with dependent errors (Wu [49]; Zhou and Shao [52]). Condition (2.6) is similar to (7) of Wu [49]. $\Vert E(\psi_{i}^{(l)}(\delta;\mathcal {F}_{i-1})|\mathcal{F}_{0})-E(\psi_{i}^{(l)}(\delta;\mathcal {F}_{i-1}^{*})|\mathcal{F}_{0}^{*}) \Vert $ measures the difference of the contribution of $\varepsilon_{0}$ and its copy $\varepsilon'_{0}$ in predicting $\psi(e_{i}+\delta)$. However, $\Vert E(\psi_{i}^{(l)}(\delta;\mathcal{F}_{i-1}^{*})|\mathcal{F}_{0})-E(\psi _{i}^{(l)}(\delta;\mathcal{F}_{i-1}^{*})|\mathcal{F}_{-1}) \Vert $ measures the contribution of $\varepsilon_{0}$ in predicting $\psi(e_{i}+\delta)$ under the given copy of $\varepsilon_{0}$: $\varepsilon'_{0}$.

If $\{\varepsilon_{i}\}$ are i.i.d., then (A6) and (A7) hold. For the other settings, (A6) and (A7) are very easily satisfied. The following proposition provides some sufficient conditions for (A6) and (A7).

### Proposition 2.1

*Let* $F_{i}(u|\mathcal{F}_{0})=P(e_{i}\leq{u}|\mathcal{F}_{0})$ *and* $f_{i}(u|\mathcal{F}_{0})$ *be the conditional distribution and density function of* $e_{i}$ *at* *u* *given* $\mathcal{F}_{0}$, *respectively*. *Let* $f_{i}(u)$ *and* $f_{i}^{*}(u)$ *be the density function of* $e_{i}$ *and* $e_{i}^{*}$, *respectively*.

1. *Let* $f_{i}(\cdot|\mathcal{F}_{i})\in{C^{l}},l\geq0$, $\omega(i)=\int_{R} \Vert f_{i}(u|\mathcal{F}_{0})-f_{i}(u|\mathcal{F}_{0}^{*}) \Vert \psi(u;\delta_{0})\,du$ *and* $\psi(u;\delta_{0})=|\psi(u+\delta_{0})|+|\psi(u-\delta_{0})|$. *If* $\sum_{i=1}^{\infty}\omega(i)<\infty$, *then* (A6) *holds*.
2. *Let*
  $$\overline{\omega}(i)= \int_{R} \bigl\Vert f_{i}^{(l)}(u| \mathcal {F}_{0})-f_{i}^{(l)}\bigl(u| \mathcal{F}_{0}^{*}\bigr) \bigr\Vert \psi(u;\delta_{0})\,du $$
  *and* $\tilde{\omega}(i)=\int_{R}|f_{i}(u)-f_{i}^{*}(u)|\psi^{(l)}(u;\delta_{0})\,du$. *If* $\sum_{i=1}^{\infty}\overline{\omega}(i)<\infty$ *and* $\sum_{i=1}^{\infty}\tilde{\omega}(i)<\infty$, *then assumption* (*A*7) *holds*.

### Proof

(1) By the conditions of (1), we have

2.7 $$\begin{aligned} &\sum_{i=1}^{\infty}\sup _{ \vert \delta \vert \leq\delta_{0}} \bigl\Vert E\bigl(\psi _{i}^{(l)}( \delta;\mathcal{F}_{i-1})|\mathcal{F}_{0}\bigr)-E\bigl( \psi_{i}^{(l)}\bigl(\delta;\mathcal{F}_{i-1}^{*} \bigr)|\mathcal{F}_{0}^{*}\bigr) \bigr\Vert \\ &\quad=\sum_{i=1}^{\infty}\sup _{ \vert \delta \vert \leq\delta_{0}} \biggl\Vert \int_{R}\psi ^{(l)}(u+\delta)\bigl[f_{i}(u| \mathcal{F}_{0})-f_{i}\bigl(u|\mathcal{F}_{0}^{*} \bigr)\bigr]\,du \biggr\Vert \\ &\qquad{}+\sup_{ \vert \delta \vert \leq\delta_{0}} \biggl\Vert \int_{R}\psi^{(l)}(u+\delta )\bigl[f_{0}(u| \mathcal{F}_{-1})-f_{0}(u|\mathcal{F}_{-1}) \bigr]\,du \biggr\Vert \\ &\quad\leq\sum_{i=1}^{\infty}\int_{R} \bigl\Vert f_{i}^{(l)}(u| \mathcal {F}_{0})-f_{i}^{(l)}\bigl(u| \mathcal{F}_{0}^{*}\bigr) \bigr\Vert \psi(u+\delta_{0})\,du \\ &\quad =\sum_{i=1}^{\infty}\omega(i)< \infty. \end{aligned}$$

Namely (A6) holds.

(2) (A7) follows from

$$\begin{aligned} &\sum_{i=1}^{\infty}\sup _{ \vert \delta \vert \leq\delta_{0}} \bigl\Vert E\bigl(\psi _{i}^{(l)} \bigl(\delta;\mathcal{F}_{i-1}^{*}\bigr)|\mathcal{F}_{0} \bigr)-E\bigl(\psi _{i}^{(l)}\bigl(\delta; \mathcal{F}_{i-1}^{*}\bigr)|\mathcal{F}_{-1}\bigr) \bigr\Vert \\ &\quad=\sum_{i=1}^{\infty}\sup _{ \vert \delta \vert \leq\delta_{0}} \biggl\Vert \int_{R}\psi ^{(l)}(u+\delta)\bigl[f_{i}^{(l)}(u| \mathcal{F}_{0})-f_{i}^{(l)}(u|\mathcal {F}_{-1})\bigr]\,du \biggr\Vert \\ &\qquad{}+\sup_{ \vert \delta \vert \leq\delta_{0}} \biggl\Vert \int_{R}\psi^{(l)}(u+\delta )\bigl[f_{0}(u| \mathcal{F}_{-1})-f_{0}\bigl(u|\mathcal{F}_{-1}^{*} \bigr)\bigr]\,du \biggr\Vert \\ &\quad \leq\sum_{i=1}^{\infty}\int_{R} \bigl\Vert f_{i}^{(l)}(u| \mathcal {F}_{0})-f_{i}^{(l)}(u| \mathcal{F}_{-1}) \bigr\Vert \psi(u+\delta_{0})\,du=\sum _{i=1}^{\infty}\overline{\omega}(i)< \infty \end{aligned}$$

and

$$\begin{aligned} &\sum_{i=1}^{\infty}\sup_{ \vert \delta \vert \leq\delta_{0}}\bigl\vert E( \psi ^{(l)}(e_{i}+\delta)-E(\psi^{(l)} \bigl(e_{i}^{*}+\delta\bigr)\bigr\vert \\ &\quad=\sum_{i=1}^{\infty}\sup _{ \vert \delta \vert \leq\delta_{0}} \biggl\vert \int_{R}\psi ^{(l)}(u+\delta) \bigl(f_{i}(u)-f_{i}^{*}(u) \bigr) \biggr\vert \\ &\quad\leq\sum_{i=1}^{\infty}\int_{R} \bigl\vert f_{i}(u)-f_{i}^{*}(u) \bigr\vert \psi^{(l)}(u;\delta _{0})\,du=\sum _{i=1}^{\infty}\tilde{\omega}(i)< \infty. \end{aligned}$$

Hence, the proposition is proved. □

Define the M-processes

$$K_{n}(\beta_{n})=\Omega_{n}(\beta_{n})-E \bigl(\Omega_{n}(\beta_{n})\bigr),\qquad \tilde{K}_{n}( \beta )=\tilde{\Omega}_{n}(\beta)-E\bigl(\tilde{\Omega}_{n}(\beta) \bigr), $$

where

$$\Omega_{n}(\beta_{n})=\sum_{i=1}^{n} \psi\bigl(e_{i}-x_{in}^{T}\beta_{n} \bigr)x_{in},\qquad \tilde{\Omega}_{n}(\beta)=\sum _{i=1}^{n}\psi\bigl(e_{i}-x_{i}^{T} \beta\bigr)x_{i}. $$

### Theorem 2.1

*Let* $\{\delta_{n},n\in{N}\}$ *be a sequence of positive numbers such that* $\delta_{n}\rightarrow\infty$ *and* $\delta _{n}{r_{n}}\rightarrow0$. *If* (A1)–(A5), *and* (A6) *and* (A7) *with* $l=0,1,\ldots,p$ *hold*, *then*

2.8 $$ \sup_{ \vert \beta \vert \leq\delta_{n}} \bigl\vert K_{n}(\beta_{n})-K_{n}(0) \bigr\vert =O_{p} \Biggl(\sqrt {\tau_{n}( \delta_{n})}\log{n}+\delta_{n}\sqrt{\sum _{i=1}^{n} \vert x_{in} \vert ^{4}} \Biggr), $$

*where*

$$\tau_{n}(\delta)=\sum_{i=1}^{n} \vert x_{in} \vert ^{2}\bigl(m^{2}\bigl( \vert x_{in} \vert \delta \bigr)+m^{2}\bigl(- \vert x_{in} \vert \delta\bigr)\bigr),\quad \delta>0. $$

### Corollary 2.1

*Assume that* (A1)–(A5), *and* (A6) *and* (A7) *with* $l=0,1,\ldots,p$ *hold*. *If* $\varphi(t)=t\varphi'(0)+O(t^{2})$ *as* $t\rightarrow0$, $\Omega(\hat{\beta}_{n})=O_{p}(r_{n})$, *then*, *for* $|\hat{\beta}_{n}|\leq\delta_{n}$,

2.9 $$ \varphi'(0)\hat{\beta}_{n}-\sum _{i=1}^{n}\psi(e_{i})x_{in} =O_{p} \bigl(\sqrt{\tau_{n}(\delta_{n})}\log{n}+ \delta_{n}^{2}r_{n} \bigr). $$

*Moreover*, *if*, *as* $t\rightarrow0$, $m(t)=O(|t|^{\lambda})$ *for some* $\lambda>0$, *then*

2.10 $$ \varphi'(0)\hat{\beta}_{n}-\sum _{i=1}^{n}\psi(e_{i})x_{in}=O_{p} \Biggl(\sqrt{\sum_{i=1}^{n} \vert x_{in} \vert ^{2+2\lambda}}\log{n}+r_{n} \Biggr). $$

### Remark 2

If $\{e_{i}\}$ i.i.d., then $|\hat{\beta}_{n}|\leq\delta_{n}$ follows from (3.2) of Rao and Zhao [42]. If $\{\varepsilon_{i}\}$ i.i.d., then $|\hat{\beta}_{n}|\leq\delta_{n}$ follows from Theorem 1 of Wu [49] and Zhou and Shao [52]. If $e_{i}=f(e_{i-1})+\varepsilon_{i}$, where the function $f:R\times{R}\rightarrow{R}$ satisfies some condition and $\{\varepsilon_{i}\}$ i.i.d., then $|\hat{\beta}_{n}|\leq\delta _{n}$ follows from Theorem 2.2 of Fan et al. [20]. If $\{\varepsilon_{i}\} $ NA, then $|\hat{\beta}_{n}|\leq\delta_{n}$ follows from Theorem 1 of Wu [47]. Therefore the condition $|\hat{\beta}_{n}|\leq\delta_{n}$ is not strong. In the paper, we do not discuss it.

### Theorem 2.2

*Assume that* (A1)–(A3), (A5), *and* (A6) *and* (A7) *with* $l=0,1,\ldots,p$ *hold*. *Let* $\lambda_{n}$ *be the minimum eigenvalue of* $\Sigma_{n}$, $b_{n}=n^{-\frac{1}{2}}(\log{n})^{{3}/{2}}(\log{\log {n}})^{{1}/{2}+\upsilon}$, $\upsilon>0$, $\tilde{n}=2^{\lceil{\log {n}}/{\log{2}}\rceil}$ *and* $q>\frac{3}{2}$. *If* $\liminf_{n\rightarrow\infty}\lambda_{n}/n>0, \sum_{i=1}^{n}|x_{i}|^{2}=O(n)$ *and* $\tilde{r}_{n}=\max_{1\leq{i}\leq{n}}|x_{i}|=O(n^{1/2}(\log{n})^{-2})$, *then*

$$\sup_{ \vert \beta \vert \leq{b_{n}}} \bigl\vert \tilde{K}_{n}(\beta)-\tilde {K}_{n}(0) \bigr\vert =O_{a.s.}(L_{\tilde{n}}+B_{\tilde{n}}), $$

*where* $L_{n}=\sqrt{\tilde{\tau}_{n}(2b_{n})}(\log_{n})^{q}$, $B_{\tilde{n}}=b_{n}(\sum_{i=1}^{n}|x_{i}|^{4})^{{1}/{2}}(\log{n})^{3/2}(\log\log {n})^{(1+\upsilon)/2}$ *and*

$$\tilde{\tau}_{n}(\delta)=\sum_{i=1}^{n} \vert x_{i} \vert ^{2}\bigl(m^{2}\bigl( \vert x_{i} \vert \delta \bigr)+m^{2}\bigl(- \vert x_{i} \vert \delta\bigr)\bigr),\quad \delta>0. $$

### Corollary 2.2

*Assume that* $\varphi(t)=t\varphi'(0)+O(t^{2})$ *and* $m(t)=O(\sqrt{t})$ *as* $t\rightarrow0$, *and* $\tilde{\Omega}_{n}=O_{a.s.}(\tilde{r}_{n})$. *Under the conditions of Theorem *2.2, *we have*:

1. $\tilde{\beta}_{n}=O_{a.s.}(b_{n})$;
2. $\varphi'(0)\Sigma_{n}\tilde{\beta}_{n}-\sum_{i=1}^{n}\psi(e_{i})x_{i} =O_{a.s.}(L_{\tilde{n}}+B_{\tilde{n}}+b_{n}^{2}\sum_{i=1}^{n}|x_{i}|^{3}+\tilde{r}_{n})$,

*where* $\tilde{\beta}_{n}$ *is the minimizer of* (1.2).

### Remark 3

From the above results, we easily obtain the corresponding conclusions of Wu [49].

From the corollary below, we only derive convergence rates of $\tilde{\beta}_{n}$. However, it is to be regretted that we cannot give laws of the iterated logarithm $n^{1/2}(\log\log{n})^{1/2}$, which is still an open problem.

### Corollary 2.3

*Under the conditions of Corollary *2.2, *we have*

$$\begin{aligned} \Sigma_{n}\tilde{\beta}_{n} ={}&O_{a.s.} \Biggl(\max \Biggl\{ \bigl(n^{1/2}( \log{n})^{3/2}(\log\log {n})^{1/2+\upsilon}\bigr),\\ &\bigl(n^{1/2}( \log{n})^{-1/4+q}(\log\log {n})^{1/4+\upsilon/2}\bigr),\Biggl(\sum _{i=1}^{n}\psi(e_{i})x_{i} \Biggr) \Biggr\} \Biggr). \end{aligned}$$

### Proof

Note that $\tilde{n}=2^{\lceil\log{n}/\log2\rceil}=O(n)$ and $m(t)=O(\sqrt{t})$ as $t\rightarrow0$; we have

$$\begin{aligned} L_{\tilde{n}}&=\sqrt{\tilde{\tau}_{n}(2b_{n})}( \log{n})^{q}=\sqrt{O\Biggl(\sum_{i=1}^{n} \vert x_{i} \vert ^{2} \vert x_{i} \vert b_{n}\Biggr)}(\log{n})^{q} \\ &=\sqrt{O\bigl(nn^{1/2}(\log{n})^{-2}n^{-1/2}( \log{n})^{3/2}(\log\log {n})^{1/2+\upsilon}\bigr)}(\log{n})^{q} \\ &=O\bigl(n^{1/2}(\log{n})^{-1/4+q}(\log\log{n})^{1/4+\upsilon/2} \bigr), \\ B_{\tilde{n}}&=O\bigl(n^{-1/2}(\log{n})^{3/2}(\log \log{n})^{1/2+\upsilon }\bigl(n\tilde{r}_{n}^{2} \bigr)^{1/2}(\log{n})^{3/2}(\log\log{n})^{(1+\upsilon)/2}\bigr) \\ &=O\bigl(n^{1/2}(\log{n}) (\log\log{n})^{1+3\upsilon/2}\bigr) \end{aligned}$$

and

$$\begin{aligned} b_{n}^{2}\sum_{i=1}^{n} \vert x_{i} \vert ^{3}&=O\bigl(n^{-1}( \log{n})^{3}(\log\log {n})^{1+2\upsilon}nn^{1/2}( \log{n})^{-2}\bigr) \\ &=O\bigl(n^{1/2}(\log{n}) (\log\log{n})^{1+2\upsilon}\bigr). \end{aligned}$$

By Corollary 2.2, we have

2.11 $$ \varphi'(0)\Sigma_{n}\tilde{\beta}_{n}=\sum _{i=1}^{n}\psi (e_{i})x_{i}+O_{a.s.} \bigl(n^{1/2}(\log{n})^{-1/4+q}(\log\log{n})^{1/4+\upsilon/2}\bigr) $$

and

2.12 $$ \Sigma_{n}\tilde{\beta}_{n}=O_{a.s.}(nb_{n})=O_{a.s.} \bigl(n^{1/2}(\log {n})^{3/2}(\log\log{n})^{1/2+\upsilon}\bigr). $$

Thus the conclusion follows from (2.11) and (2.12). □

## Applications

In the following three subsections, we shall investigate some applications of our results. In Sect. 3.1, we consider that $\varepsilon _{i}$ is a *m*-dependent random variable sequence. We shall investigate that $\{\varepsilon_{i}\}$ are $(\varepsilon,\psi)$-weakly dependent in Sect. 3.2, and martingale difference errors $\{\varepsilon_{i}\}$ in Sect. 3.3.

### *m*-dependent process

In the subsection, we shall firstly show that the *m*-dependent sequence satisfies conditions (A6) and (A7) and secondly obtain the asymptotic normal distribution and strong convergence rates for M-estimators of the parameter. Koul [30] discussed the asymptotic behavior of a class of M-estimators in the model (1.1) with long range dependence errors $e_{i}=g(\varepsilon_{i})$, where $\varepsilon_{i}$ i.i.d. Here we assume that $\varepsilon_{i}$ is a *m*-dependent sequence, of which the definition was given by Example 2.8.1 in Lehmann [32]. For *m*-dependent sequences or processes, there are some results (e.g., see Hu et al. [27], Romano and Wolf [43] and Valk [46]).

#### Proposition 3.1

*Let* $\varepsilon_{i}$ *in* (1.4) *be a* *m*-*dependent sequence*. *Then* (A6) *and* (A7) *hold*.

#### Proof

Note that $\varepsilon_{i}$ is a *m*-dependent sequence, we have

3.1 $$\begin{aligned} &\sum_{i=1}^{\infty}\sup_{ \vert \delta \vert \leq\delta_{0}} \bigl\Vert E\bigl(\psi _{i}^{(l)}(\delta; \mathcal{F}_{i-1})|\mathcal{F}_{0}\bigr)-E\bigl( \psi_{i}^{(l)}\bigl(\delta;\mathcal{F}_{i-1}^{*} \bigr)|\mathcal{F}_{0}^{*}\bigr) \bigr\Vert \\ &\quad=\sum_{i=1}^{\infty}\sup _{ \vert \delta \vert \leq\delta_{0}} \bigl\Vert E\bigl(\psi ^{(l)}(e_{i}+ \delta)|\mathcal{F}_{0}\bigr)-E\bigl(\psi^{(l)} \bigl(e_{i}^{*}+\delta\bigr)|\mathcal {F}_{0}^{*}\bigr) \bigr\Vert \\ &\qquad{}+\sup_{ \vert \delta \vert \leq\delta_{0}} \bigl\Vert E\bigl(\psi^{(l)}(e_{i}+ \delta)|\mathcal {F}_{-1}\bigr)-E\bigl(\psi^{(l)} \bigl(e_{i}^{*}+\delta\bigr)|\mathcal{F}_{-1}\bigr) \bigr\Vert \\ &\quad=\sum_{i=1}^{\infty}\sup _{ \vert \delta \vert \leq\delta_{0}} \bigl\Vert E\bigl(\psi ^{(l)}(e_{i}+ \delta)\bigr)-E\bigl(\psi^{(l)}\bigl(e_{i}^{*}+\delta\bigr) \bigr) \bigr\Vert \\ &\quad =0< \infty \end{aligned}$$

and

3.2 $$\begin{aligned} &\sum_{i=1}^{\infty}\sup_{ \vert \delta \vert \leq\delta_{0}} \bigl\Vert E\bigl(\psi _{i}^{(l)}\bigl(\delta; \mathcal{F}_{i-1}^{*}\bigr)|\mathcal{F}_{0}\bigr)-E\bigl(\psi _{i}^{(l)}\bigl(\delta;\mathcal{F}_{i-1}^{*}\bigr)| \mathcal{F}_{-1}\bigr) \bigr\Vert \\ &\quad=\sum_{i=1}^{\infty}\sup _{ \vert \delta \vert \leq\delta_{0}} \bigl\Vert E\bigl(\psi ^{(l)} \bigl(e_{i}^{*}+\delta\bigr)|\mathcal{F}_{0}\bigr)-E\bigl( \psi^{(l)}\bigl(e_{i}^{*}+\delta\bigr)|\mathcal {F}_{-1}\bigr) \bigr\Vert \\ &\qquad{}+\sup_{ \vert \delta \vert \leq\delta_{0}} \bigl\Vert E\bigl(\psi^{(l)}(e_{0}+ \delta)|\mathcal {F}_{-1}\bigr)-E\bigl(\psi^{(l)} \bigl(e_{0}^{*}+\delta\bigr)|\mathcal{F}_{-1}\bigr) \bigr\Vert \\ &\quad=\sum_{i=1}^{\infty}\sup _{ \vert \delta \vert \leq\delta_{0}} \bigl\Vert E\psi ^{(l)}(e_{0}+ \delta)-E\psi^{(l)}\bigl(e_{i}^{*}+\delta\bigr) \bigr\Vert \\ &\quad=0< \infty. \end{aligned}$$

Therefore, (A6) and (A7) follow from (3.1), (3.2) and $E\psi ^{(l)}(e_{i}+\delta)=E\psi^{(l)}(e_{i}^{*}+\delta)$. □

#### Corollary 3.1

*Assume that* (A1)–(A5) *hold*. *If* $\varphi(t)=t\varphi'(0)+O(t^{2})$ *and* $m(t)=O(|t|^{\lambda})$ *for some* $\lambda>0$ *as* $t\rightarrow0$, $\Omega (\hat{\beta}_{n})=0$ *and* $0<\sigma_{\psi}^{2}=E[\psi(e_{i})]^{2}<\infty$, *then*

$$n^{-1/2}\hat{\beta}_{n}/\bigl(\bigl[\varphi'(0) \bigr]^{-1}\sigma_{\psi}\bigr)\rightarrow {N}(0,I_{p}),\quad n \rightarrow\infty. $$

*In order to prove Corollary *3.1, *we give the following lemmas*.

#### Lemma 3.1

(Lehmann [32])

*Let* $\{\xi_{i},i\geq1\}$ *be a stationary* *m*-*dependent sequence of random variables with* $E\xi_{i}=0$ *and* $0<\sigma ^{2}=\operatorname{Var}(\xi_{i})<\infty$, *and* $T_{n}=\sum_{i=1}^{n}\xi_{i}$. *Then*

$$n^{-1/2}T_{n}/\tau\rightarrow{N}(0,1), $$

*where* $\tau^{2}=\lim_{n\rightarrow\infty}\operatorname{Var}(n^{-1/2}T_{n})=\sigma ^{2}+2\sum_{i=2}^{m+1}\operatorname{Cov}(\xi_{1},\xi_{i})$.

Using the argument of Lemma 3.1, we easily obtain the following result. Here we omit the proof.

#### Lemma 3.2

*Let* $\{\xi_{i},i\geq1\}$ *be a stationary* *m*-*dependent sequence of random variables with* $E\xi_{i}=0$ *and* $0<\sigma_{i}^{2}=\operatorname{Var}(\xi _{i})<\infty$, *and* $T_{n}=\sum_{i=1}^{n}\xi_{i}$. *Then*

$$n^{-1/2}T_{n}/\tau\rightarrow{N}(0,1), $$

*where* $\tau^{2}=\operatorname{Var}(n^{-1/2}T_{n})=n^{-1}\sum_{i=1}^{n}\sigma _{i}^{2}+2n^{-1}\sum_{i=2}^{m+1}(n-i)\operatorname{Cov}(\xi_{1},\xi_{i})$.

#### Proof of Corollary 3.1

By (2.10), we have

3.3 $$ n^{-1/2}\hat{\beta}_{n}=n^{-1/2}\bigl[ \varphi'(0)\bigr]^{-1}\sum_{i=1}^{n} \psi (e_{i})x_{in}+ O_{p}\bigl(n^{-1/2}r_{n}^{\lambda}\log{n}\bigr). $$

Since $\{\xi_{i},i\geq1\}$ is a stationary *m*-dependent sequence, so is $\{[\varphi'(0)]^{-1}\psi({e}_{i}){x}_{in},i\geq1\}$. Let $u\in{R}^{p}$, $|u|=1$. Then $E (u^{T}[\varphi'(0)]^{-1}\psi({e}_{i}){x}_{in} )=0$ and

$$\sigma_{i}^{2}=E \bigl(u^{T}\bigl[ \varphi'(0)\bigr]^{-1}\psi(e_{i}){x}_{in} \bigr)^{2}=\bigl[\varphi'(0)\bigr]^{-2}u^{T}x_{in} x^{T}_{in}uE\bigl[\psi({e}_{i}) \bigr]^{2}. $$

Therefore, by $r_{n}=o(1)$ and $0<\sigma_{\psi}^{2}={E}[\psi({e}_{i})]^{2}<\infty $, we have

3.4 $$\begin{aligned} \tau^{2}={}&n^{-1}\sum_{i=1}^{n} \bigl[\varphi '(0)\bigr]^{-2}u^{T}x_{in}x^{T}_{in}u{E} \bigl[\psi({e}_{i})\bigr]^{2} \\ &{}+2n^{-1}\sum_{i=2}^{m+1}(n-i)\operatorname{Cov} \bigl({u}^{T}\bigl[\varphi'(0)\bigr]^{-1}\psi (e_{1})x_{1n},{u}^{T}\bigl[\varphi'(0) \bigr]^{-1}\psi(e_{i})x_{in}\bigr) \\ ={}&\bigl[\varphi'(0)\bigr]^{-2}n^{-1} \Biggl\{ \sum_{i=1}^{n}{E}\bigl[\psi ({e}_{i})\bigr]^{2}+2\sum_{i=2}^{m+1}(n-i)u^{T}x_{in}x^{T}_{in}u\operatorname{Cov} \bigl(\psi ({e}_{1}),\psi({e}_{i}) \bigr) \Biggr\} \\ \rightarrow{}&\bigl[\varphi'(0)\bigr]^{-2} \sigma_{\psi}^{2}. \end{aligned}$$

Thus the corollary follows from Lemma 3.2, (3.3) and (3.4). □

#### Corollary 3.2

*Assume that* (A1)–(A5) *hold*. *If* $\varphi(t)=t\varphi '(0)+O(t^{2})$ *and* $m(t)=O(\sqrt{t})$ *as* $t\rightarrow0$, *and* $\tilde{\Omega}_{n}(\tilde{\beta}_{n})=O_{a.s.}(\tilde{r}_{n}),0<\sigma_{\psi}^{2}={E}[\psi ({e}_{i})]^{2}<\infty$, *then*

$$\tilde{\beta}_{n}=O_{a.s.} \bigl(n^{-1/2}( \log{n})^{3/2}(\log\log {n})^{1/2+\upsilon} \bigr). $$

#### Proof

The corollary follows from Proposition 3.1 and Corollary 2.2. □

### $(\varepsilon,\psi)$-weakly dependent process

In the subsection, we assume that $\{\varepsilon_{i}\}$ are $(\varepsilon ,\psi)$-weakly dependent (Doukhan and Louhichi [14] and Dedecker et al. [11]) random variables. In 1999, Doukhan and Louhichi proposed a new idea of $(\varepsilon,\psi)$-weakly dependence which focuses on covariance rather than the total variation distance between joint distributions and the product of the corresponding marginal. It has been shown that this concept is more general than mixing and includes, under natural conditions on the process parameters, essentially all classes of processes of interest in statistics. Therefore, many researchers are interested in the $(\varepsilon,\psi)$-weakly dependent and related possesses, and one obtained lots of sharp results. For example, Doukhan and Louhichi [14], Dedecker and Doukhan [10], Dedecker and Prieur [12], Doukhan and Neumann [16], Doukhan and Wintenberger [17], Bardet et al. [3], Doukhan and Wintenberger [18], Doukhan et al. [13]. However, a few people (only Hwang and Shin [29], Nze et al. [40]) investigated regression models with $(\varepsilon,\psi)$-weakly dependent errors. Nobody has investigated a robust estimate for the regression model with $(\varepsilon,\psi )$-weakly dependent errors. To give the definition of the $(\varepsilon,\psi)$-weakly dependence, let us consider a process $\xi=\{\xi_{n},n\in{Z}\}$ with values in a Banach space $(\mathcal{E}, \Vert \cdot \Vert )$. For $h:\mathcal{E}^{u}\rightarrow {R}$, $u\in{N}$, we define the Lipschitz modulus of *h*,

3.5 $$ \mathrm{Lip} h=\sup_{y\neq{x}} \bigl\vert h(y)-h(x) \bigr\vert / \Vert y-x \Vert _{1}, $$

where we have the $l_{1}$-norm, i.e., $\Vert (y_{1},y_{2},\ldots,y_{u}) \Vert _{1}=\sum_{i=1}^{u}|y_{i}|$.

#### Definition 1

(Doukhan and Louhich [14])

A process $\xi=\{\xi _{n},n\in{Z}\}$ with values in $R^{d}$ is called a $(\varepsilon,\psi )$-weakly dependent process if, for some classes of functions $\mathcal {E}^{u},\mathcal{E}^{v}\rightarrow{R},F_{u},G_{v}$:

$$\begin{aligned} \varepsilon(r)&=\sup_{u,v}\sup_{s_{1}\geq{s}_{2}\geq\cdots\geq {s}_{u},t_{1}\geq{t}_{2}\geq\cdots\geq{t}_{v},r=t_{1}-s_{u}}\sup _{f\in {F_{u}},g\in{G_{v}}}\frac{|\operatorname{Cov}(f(\xi_{s_{1}},\xi_{s_{2}},\ldots,\xi_{s_{u}}),g(\xi _{t_{1}},\xi_{t_{2}},\ldots,\xi_{t_{v}}))|}{\Psi(f,g)}\\ &\rightarrow0 \end{aligned}$$

as $r\rightarrow\infty$.

According to the definition, mixing sequences ($\alpha,\rho,\beta ,\varphi$-mixing), associated sequences (positively or negatively associated), Gaussian sequences, Bernoulli shifts and Markovian models or time series bootstrap processes with discrete innovations are $(\varepsilon,\psi)$-weakly dependent (Doukhan et al. [15]).

From now on, assume that the classes of functions contain functions bounded by 1. Distinct functions Ψ yield $\eta,\theta,\kappa$ and a *λ* weak dependence of the coefficients as follows (Doukhan et al. [15]):

3.6 $$\begin{aligned} \Psi(f,g)= \textstyle\begin{cases} u\mathrm{Lip}f+v\mathrm{Lip}g & \text{then denote }\varepsilon(r)=\eta(r), \\ v\mathrm{Lip}g & \text{then denote }\varepsilon(r)=\theta(r), \\ uv\mathrm{Lip}f\cdot \mathrm{Lip}g & \text{then denote } \varepsilon(r)=\kappa(r), \\ u\mathrm{Lip}f+v\mathrm{Lip}g+uv\mathrm{Lip}f\cdot \mathrm{Lip}g & \text{then denote } \varepsilon(r)=\lambda (r),\\ u\mathrm{Lip}f+v\mathrm{Lip}g+uv\mathrm{Lip}f\cdot \mathrm{Lip}g+u+v & \text{then denote } \varepsilon (r)=\omega(r). \end{cases}\displaystyle \end{aligned}$$

In Corollary 3.3, we only consider *λ* and *η*-weakly dependence. Let $\{\varepsilon_{i}\}$ be *λ* or *η*-weakly dependent, and assume that *g* satisfies: for each $s\in{Z}$, if $x,y\in {R^{Z}}$ satisfy $x_{i}=y_{i}$ for each index $i\neq{s}$

3.7 $$ \bigl\vert g(x)-g(y) \bigr\vert \leq b_{s}\Bigl(\sup _{i\neq{s}} \vert x_{i} \vert ^{l}\vee1 \Bigr) \vert x_{s}-y_{s} \vert . $$

#### Lemma 3.3

(Dedecker et al. [11])

*Assume that* *g* *satisfies the condition* (3.7) *with* $l\geq0$ *and some sequence* $b_{s}\geq0$ *such that* $\sum_{s}|s|b_{s}<\infty$. *Assume that* $E|\varepsilon_{0}|^{m'}<\infty $ *with* $lm< m'$ *for some* $m>2$. *Then*:

1. *If the process* $\{\varepsilon_{i},i\in{Z}\}$ *is* *λ*-*weakly dependent with coefficients* $\lambda_{\varepsilon}(r)$, *then* $e_{n}$ *is* *λ*-*weakly dependent with coefficients*
  3.8 $$ \lambda_{e}(k)=c\inf_{r\leq[k/2]}\biggl(\sum _{i\geq{r}}b_{i}\biggr)\vee \bigl[(2r+1)^{2} \lambda_{\varepsilon}(k-2r)^{\frac{m'-1-l}{m'-1+l}}\bigr]. $$
2. *If the process* $\{\varepsilon_{i},i\in{Z}\}$ *is* *η*-*weakly dependent with coefficients* $\eta_{\varepsilon}(r)$, *then* $e_{n}$ *is* *η*-*weakly dependent and there exists a constant* $c>0$ *such that*
  $$ \eta_{e}(k)=c\inf_{r\leq[k/2]}\biggl(\sum _{i\geq{r}}b_{i}\biggr)\vee \bigl[(2r+1)^{1+\frac{1}{m'-1}} \eta_{\varepsilon}(k-2r)^{\frac{m'-2}{m'-1}}\bigr]. $$

#### Lemma 3.4

(Bardet et al. [3])

*Let* $\{\xi_{n},n\in{Z}\}$ *be a sequence of* $R^{k}$-*valued random variables*. *Assume that there exists some constant* $C>0$ *such that* $\max_{1\leq{i}\leq{k}} \Vert \xi_{i} \Vert _{p}\leq{C},p\geq1$. *Let* *h* *be a function from* $R^{k}$ *to* *R* *such that* $h(0)=0$ *and for* $x,y\in{R^{k}}$, *there exist* *a* *in* $[1,p]$ *and* $c>0$ *such that*

3.9 $$ \bigl\vert h(x)-h(y) \bigr\vert \leq{c} \vert x-y \vert \bigl(1+ \vert x \vert ^{\alpha-1}+ \vert y \vert ^{\alpha-1}\bigr). $$

*Now we define the sequence* $\{\zeta_{n},n\in{Z}\}$ *by* $\zeta_{n}=h(\xi_{n})$. *Then*:

(1) *If the process* $\{\xi_{i},i\in{Z}\}$ *is* *λ*-*weakly dependent with coefficients* $\lambda_{\xi}(r)$, *then* $\{\zeta_{n},n\in{Z}\}$ *is also with coefficients*

3.10 $$ \lambda_{\zeta}(r)=O\bigl(\lambda_{\xi}^{\frac{p-a}{p+a-2}}(r) \bigr). $$

(2) *If the process* $\{\xi_{i},i\in{Z}\}$ *is* *ζ*-*weakly dependent with coefficients* $\eta_{\xi}(r)$, *so is* $\{\zeta_{n},n\in{Z}\}$ *with coefficients* $\eta_{\zeta}(r)=O(\eta_{\xi}^{\frac{p-a}{p-1}}(r))$.

#### Lemma 3.5

(Dedecker et al. [11])

*Let* $\{\xi_{i},i\in{Z}\}$ *be a centered and stationary real*-*valued sequence with* $E|\xi_{0}|^{2+\varsigma }<\infty$, $\varsigma>0$, $\sigma^{2}=\sum_{k\in{Z}}\operatorname{Cov}(\xi_{0},\xi _{k})$ *and* $S_{n}=\sum_{i=1}^{n}\xi_{i}$. *If* $\lambda_{\xi}(r)=O(r^{-\lambda})$ *for* $\lambda>4+2/\varsigma$, *then* $n^{-1/2}S_{n}\rightarrow{N}(0,\sigma^{2})$ *as* $n\rightarrow\infty$.

#### Corollary 3.3

*Let* $\{\varepsilon_{i}\}$ *be* *λ*-*weakly dependent with coefficients* $\lambda_{\varepsilon}(r)=O(\exp(-r\lambda))$ *for some* $\lambda>0$, *and* $b_{i}=O(\exp(-ib))$ *for some* $b>0$. *Assume that* $\psi(0)=0$, *and*, *for* $x,y\in{R}$, *there exists a constant* $c>0$ *such that*

3.11 $$ \bigl\vert \psi(x)-\psi(y) \bigr\vert \leq{c} \vert x-y \vert . $$

*Under the conditions of Corollary *2.1, *we have*

3.12 $$ \varphi'(0)n^{-1/2}T_{n}\rightarrow{N}(0,\Sigma)\quad \textit{as } n\rightarrow \infty, $$

*where* $\Sigma=\sum_{i=1}^{n}{x}_{1n}\operatorname{Cov}(\psi(e_{1}),\psi(e_{i}))x_{in}^{T}$.

#### Proof

Note that $\{\varepsilon_{i}\}$ is *λ*-weakly dependent. By Lemma 3.3, we find that $\{e_{i}\}$ is *λ*-weakly dependent with coefficients

3.13 $$ \lambda_{e}(r)=O\biggl(r^{2}\exp\biggl(-\lambda{r} \frac{b(m'-1-l)}{b(m'-1+l+2\alpha (m'-1-l))}\biggr)\biggr),\quad \alpha>0 $$

from (3.8) and Proposition 3.1 in Chap. 3 (Dedecker et al. [11]).

Let $u\in{R}^{p}$, $|u|=1$, and $\zeta_{i}=h(e_{i})=u\psi(0)x_{in}=0$. Then $h(0)=0=u\psi(0)x_{in}=0$. Choose $p=2,a=1$, in (3.9), and by (3.11), we have

3.14 $$ \bigl\vert h(x)-h(y) \bigr\vert = \vert x_{in} \vert \bigl\vert \psi(x)-\psi(y) \bigr\vert \leq{c} \vert x-y \vert $$

for $x, y\in{R}$ and $c>0$. Therefore, by Lemma 3.4, $\{\zeta_{i},i\in{N}\}$ is *λ*-weakly dependent with coefficients

3.15 $$ \lambda_{\zeta}(r)=O\bigl(r_{n}^{uv} \lambda_{e}^{\frac {p-a}{p+a-2}}(r)\bigr)=O\bigl(r_{n}^{uv} \lambda_{e}(r)\bigr). $$

By Corollary 2.1, we have

3.16 $$ \varphi'(0)n^{-1/2}\hat{\beta}_{n}= n^{-1/2}\sum_{i=1}^{n} \psi(e_{i})x_{in}+o_{p}(1). $$

By (3.13) and (3.15), there exist $b>0,a>0,l\geq0$ and $m'>lm$ for some $m>2$ such that

3.17 $$ \lambda_{\zeta}(r)=O\biggl(r_{n}^{uv}r^{2} \exp\biggl(-\lambda{r}\frac {b(m'-1-l)}{b(m'-1+l)+2\alpha(m^{\prime}-1-l)}\biggr)\biggr)=O\bigl(r^{-\lambda} \bigr) $$

for enough large *r* and $\lambda>4+2/\varsigma$ with $\varsigma>0$.

By Lemma 3.5 and (3.16)–(3.17), we have

$$\varphi'(0)n^{-1/2}uT_{n}\rightarrow{N}\bigl(0, \sigma^{2}\bigr), $$

where $\sigma^{2}=\sum_{i=1}^{n}u^{T}x_{1n}\operatorname{Cov}(\psi(e_{1}),\psi (e_{i}))x_{in}^{T}u$. Using the Cramer device, we complete the proof of Corollary 3.3. □

#### Lemma 3.6

(Dedecker et al. [11])

*Suppose that* $\{\xi_{i},1\leq {i}\leq{n}\}$ *are stationary real*-*valued random variables with* $E\xi _{i}=0$ *and* $P(|\xi_{i}|\leq{M}<\infty)=1$ *for all* $i=1,2,\ldots,n$. *Let* $\Psi:N^{2}\rightarrow{N}$ *be one of the following functions*:

3.18 $$\begin{aligned} \begin{aligned} &\Psi(u,v)=2v,\qquad \Psi(u,v)=u+v, \qquad \Psi(u,v)=uv,\\ &\Psi(u,v)=\alpha(u+v)+(1-\alpha)uv, \end{aligned} \end{aligned}$$

*for some* $0<\alpha<1$. *We assume that there exist constants* $K,L_{1},L_{2}<\infty,\mu\geq0$ *and a nonincreasing sequence of real coefficients* $\{\rho(n),n\geq0\}$ *such that*, *for all* *u*-*tuples* $(s_{1},\ldots,s_{u})$ *and all* *v*-*tuples* $(t_{1},\ldots,t_{v})$ *with* $1\leq {s_{1}}\leq\cdots\leq{s_{u}}\leq{t_{1}}\leq\cdots\leq{t_{v}}\leq{n,}$ *the following inequality is fulfilled*:

3.19 $$ \bigl\vert \operatorname{Cov}(\xi_{s_{1}},\ldots,\xi_{s_{u}}; \xi_{t_{1}},\ldots,\xi_{t_{v}}) \bigr\vert \leq {K}^{2}M^{u+v-2}\Psi(u,v)\rho(t_{1}-s_{u}), $$

*where*

3.20 $$ \sum_{s=0}^{\infty}(s+1)^{k} \rho(s)\leq{L_{1}}L_{2}^{k}(k!)^{\mu},\quad \forall {k}\geq0. $$

*Let* $S_{n}=\sum_{i=1}^{n}\xi_{i}$ *and* $\sigma_{n}^{2}=\operatorname{Var}(\sum_{i=1}^{n}\xi_{i})$. *If* $\sigma^{2}=\lim_{n\rightarrow\infty}\sigma_{n}^{2}/n>0$, *then*

3.21 $$ \limsup_{n\rightarrow\infty}\frac{ \vert S_{n} \vert }{\sigma(2n\log\log {n})^{1/2}}\leq1. $$

#### Corollary 3.4

*Let* $\{\varepsilon_{i}\}$ *be* *η*-*weakly dependent with coefficients* $\eta_{\varepsilon}(r)=O(\exp(-r\eta))$ *for some* $\eta >0$, *and* $b_{i}=O(\exp(-ib))$ *for some* $b>0$. *Assume that* $\psi(0)=0$ *and* (3.11) *hold*. *Under the conditions of Corollary *2.2 *with* $\tilde {r}_{n}=O(n^{1/2}(\log{n})^{-2})$ *replaced by* $0<\min_{1\leq{i}\leq {n}}|x_{ij}|<\max_{1\leq{i}\leq{n}}|x_{ij}|<\infty$, *and* $0<\sigma_{\psi}^{2}=E\psi^{2}(e_{i})<\infty$, *we have*:

1. *for* $3/2< q\leq7/4, \Sigma_{n}\tilde{\beta}_{n}=O_{a.s.}(nb_{n})=O_{a.s.}(n^{1/2}(\log{n})^{3/2}(\log\log {n})^{1/2+\upsilon})$;
2. *for* $q\geq7/4, \Sigma_{n}\tilde{\beta}_{n}=O_{a.s.}(n^{1/2}(\log {n})^{-1/4+q}(\log\log{n})^{1/4+2/\upsilon})$.

#### Proof

Let $\xi_{i}=\psi(e_{i})x_{ij},j=1,\ldots,p$. Then for $\forall \mu_{n}\rightarrow\infty$ as $n\rightarrow\infty$

3.22 $$ P\bigl\{ \bigl\vert \psi(e_{i})x_{ij} \bigr\vert > \mu_{n}\bigr\} \leq\frac{E \vert \psi(e_{i})x_{ij} \vert ^{2}}{\mu _{n}^{2}}=\frac{\sigma_{\psi}^{2}\max_{1\leq{i}\leq{n}} \vert x_{ij}^{2} \vert }{\mu _{n}^{2}}\rightarrow0. $$

Therefore, there exists some $0< M<\infty$ such that

3.23 $$ P\bigl\{ \bigl\vert \psi(e_{i})x_{ij} \bigr\vert \leq{M}\bigr\} =1. $$

Similar to the proofs of (3.13) and (3.15), we easily obtain

3.24 $$ \eta_{\zeta}(r)=O\bigl(\tilde{r}_{n}^{uv} \eta_{e}^{\frac{p-a}{p+a-2}}(r)\bigr)=O\bigl(\tilde {r}_{n}^{uv} \eta_{e}(r)\bigr), $$

where

3.25 $$ \eta_{e}(r)=O\biggl(r^{\frac{m'-1-l}{m'-1}}\exp\biggl(-\eta{r} \frac {b(m'-2)}{b(m'-1)+2\eta(m'-2)}\biggr)\biggr). $$

By (3.24) and (3.25), we have

3.26 $$\begin{aligned} &\bigl\vert \operatorname{Cov}(\xi_{s_{1}},\ldots,\xi_{s_{u}}; \xi_{t_{1}},\ldots,\xi_{t_{v}}) \bigr\vert \\ &\quad\leq (u+v) \eta_{\zeta}(r)\leq(u+v)\tilde{r}_{n}^{uv} \eta_{e}(r) \\ &\quad\leq(u+v)\tilde{r}_{n}^{uv}r^{\frac{m'-1-l}{m'-1}}\exp\biggl(- \eta{r}\frac {b(m'-2)}{b(m'-1)+2\eta(m'-2)}\biggr). \end{aligned}$$

Let $\Psi(u,v)=u+v,K^{2}={r}_{n}^{uv}M^{1(u+v-2)}$ and

3.27 $$ \rho(s)=r^{\frac{m'-1-l}{m'-1}}\exp\biggl(-\eta{r}\frac{b(m'-2)}{b(m'-1)+2\eta (m'-2)}\biggr). $$

Thus (3.19) holds. Since $\lim_{s\rightarrow\infty}\ln (s+1)/s=0$, there exist $b>0,\eta>0,l\geq0$ and $m'>lm$ for some $m>2$ and $m'>2$ such that

3.28 $$ \exp \biggl(-\eta{s}\frac{b(m'-2)}{b(m'-1)+2\eta(m'2)} \biggr)\leq (s+1)^{-(2+k)},\quad \forall k\geq0. $$

Thus

3.29 $$\begin{aligned} &\sum_{s=0}^{\infty}(s+1)^{k} \rho(s)\leq\sum_{s=0}^{\infty}(s+1)^{k+\frac{m'-1-l}{m'-1}}\exp\biggl(-\eta{s}\frac{b(m'-2)}{b(m'-1)+2\eta (m'-2)}\biggr) \\ &\phantom{\sum_{s=0}^{\infty}(s+1)^{k} \rho(s)}\leq\sum_{s=0}^{\infty}(s+1)^{-2+\frac{m'-1-l}{m'-1}}< \infty, \end{aligned}$$

3.30 $$\begin{aligned} &\sigma^{2}=\lim_{n\rightarrow\infty}\Biggl\{ n^{-1}\sum _{i=1}^{n}E\psi^{2}(e_{i})x_{ij}^{2}+n^{-1} \sum_{i,k=1;i\neq k}^{n}x_{ij}x_{kj}\operatorname{Cov} \bigl(\psi(e_{i}),\psi(e_{k})\bigr)\Biggr\} \\ &\phantom{\sigma^{2}}=\lim_{n\rightarrow\infty}\Biggl\{ n^{-1}\sum _{i=1}^{n}E\psi ^{2}(e_{i})x_{ij}^{2}+ \frac{n-1}{2}O\bigl(x_{ij}^{2}\bigr)\sum _{i=1}^{n-1}(n-i)\operatorname{Cov}\bigl(\psi(e_{1}), \psi(e_{i+1})\bigr)\Biggr\} \\ &\phantom{\sigma^{2}}=\sigma_{\psi}^{2}\bar{x}_{\cdot j}>0. \end{aligned}$$

By Lemma 3.6 and Corollary 2.3, we have

3.31 $$\begin{aligned} \Sigma_{n}\tilde{\beta}_{n}&=O_{a.s.}\bigl((2n\log \log n)^{1/2}\bigr)+O_{a.s.}\bigl(n^{1/2}(\log n)^{-1/4+q}(\log\log n)^{1/4+\upsilon /2}\bigr) \\ &=O_{a.s.}\bigl(n^{1/2}(\log n)^{-1/4+q}(\log\log n)^{1/4+\upsilon/2}\bigr). \end{aligned}$$

Therefore, by Corollary 2.3, (3.23) and (3.31), we complete the proof of Corollary 3.4. □

### Linear martingale difference processes

In the subsection, we will investigate martingale difference errors $\{ \varepsilon_{i}\}$. We shall provide some sufficient conditions for (A6) and (A7) and give the central limit theorem and strong convergence rates.

Let $\{\varepsilon_{i}\}$ be a martingale difference sequence, and $a_{j}$ be real numbers such that $e_{i}=\sum_{j=0}^{\infty}a_{j}\varepsilon _{i-j}$ exists. It is well known that the theory of martingales provides a natural unified method for dealing with limit theorems. Under its influence, there is great interest in the martingale difference. Liang and Jing [34] were concerned with the partial linear model under the linear com of martingale differences and obtained asymptotic normality of the least squares estimator of the parameter. Nelson [39] has given conditions for the pointwise consistency of weighted least squares estimators from multivariate regression models with martingale difference errors. Lai [31] investigated stochastic regression models with martingale difference sequence errors and obtained strong consistency and asymptotic normality of the least squares estimate of the parameter.

Let $F_{\varepsilon}$ be the distribution function of $\varepsilon_{0}$ and let $f_{\varepsilon}$ be its density.

#### Proposition 3.2

*Suppose that* $E\varepsilon_{0}=0,\varepsilon_{0}\in L^{4/(2-\gamma)}$, $\kappa_{\gamma}=\int_{R}\psi^{2}(u)\omega_{-\gamma }(du)<\infty,1<\gamma<2$ *and* $\sum_{k=0}^{p}\int_{R}|f_{\varepsilon}^{(k)}(v)|^{2}\omega_{\gamma}(dv)<\infty$, *where* $\omega_{\gamma}(dv)=(1+|v|)^{\gamma}$. *If* $\sum_{j=0}^{\infty}|a_{j}|<\infty$, *then* $\sum_{i=0}^{\infty}\omega(i)<\infty,\sum_{i=0}^{\infty}\bar{\omega}(i)<\infty$ *and* $\sum_{i=0}^{\infty}\tilde{\omega}(i)<\infty$.

#### Proof

Let $Z_{n}=\sum_{j=0}^{\infty}a_{j}\varepsilon _{n-j},Z_{n}^{*}=Z_{n}-a_{n}\varepsilon_{0}-a_{n}\varepsilon'$, and

3.32 $$ R_{n}= \int_{R}\bigl[f_{\varepsilon}(t-U_{n})-f_{\varepsilon}(t-U_{n}-a_{0} \varepsilon _{n})\bigr]^{2}\omega_{\gamma}(dt), $$

where $U_{n}=Z_{n}-a_{n}\varepsilon_{0}$. By the Schwartz inequality, we have

3.33 $$\begin{aligned} \omega^{2}(n)&=\biggl( \int_{R} \bigl\Vert f_{\varepsilon}(t-Z_{n})-f_{\varepsilon}\bigl(t-Z_{n}^{*}\bigr) \bigr\Vert \bigl(1+ \vert t \vert \bigr)^{\gamma}\cdot\psi(t;\varepsilon_{0}) \bigl(1+ \vert t \vert \bigr)^{-\gamma}\,dt\biggr)^{2} \\ &\leq \int_{R}\psi^{2}(t;\varepsilon_{0}) \omega_{-\gamma}(dt)\cdot \int_{R} \bigl\Vert f_{\varepsilon}(t-Z_{n})-f_{\varepsilon}\bigl(t-Z_{n}^{*}\bigr) \bigr\Vert ^{2}\omega_{\gamma}(dt) \\ &=\kappa_{\gamma}\int_{R} \bigl\Vert f_{\varepsilon}(t-Z_{n})-f_{\varepsilon}\bigl(t-Z_{n}^{*}\bigr) \bigr\Vert ^{2}\omega_{\gamma}(dt) \leq CE(R_{n}). \end{aligned}$$

Note that

3.34 $$ f_{\varepsilon}(t-U_{n})-f_{\varepsilon}(t-U_{n}-a_{n} \varepsilon_{0})= \int _{0}^{a_{n}\varepsilon_{0}}f_{\varepsilon}^{\prime}(t-U_{n}-v)\,dv $$

and

3.35 $$\begin{aligned} \int_{R}\bigl[f^{\prime}_{\varepsilon}(t-u) \bigr]^{2}\omega_{\gamma}(dt)&=\bigl(1+ \vert u \vert \bigr)^{\gamma}\int _{R}\bigl[f^{\prime}_{\varepsilon}(v) \bigr]^{2}\bigl(1+ \vert u \vert \bigr)^{-\gamma}\bigl(1+ \vert u+v \vert \bigr)^{\gamma}\,dv \\ &\leq\bigl(1+ \vert u \vert \bigr)^{\gamma}\int_{R}\bigl[f^{\prime}_{\varepsilon}(v) \bigr]^{2}\omega_{\gamma}(dv)\leq I_{1}\bigl(1+ \vert u \vert \bigr)^{\gamma}. \end{aligned}$$

Let $I_{k}=\int_{R}[f^{(k)}_{\varepsilon}(v)]^{2}\omega_{\gamma}(dv)$. By the Schwartz inequality, we have

3.36 $$\begin{aligned} R_{n}&\leq \int_{R} \biggl\vert \int_{0}^{a_{n}\varepsilon_{0}}1^{2}\,dv\cdot \int_{0}^{a_{n}\varepsilon _{0}}\bigl[f_{\varepsilon}^{\prime}(t-U_{n}-v) \bigr]^{2}\,dv \biggr\vert \omega_{\gamma}(dt) \\ &\leq \vert a_{n}\varepsilon_{0} \vert \int_{0}^{a_{n}\varepsilon_{0}}I_{1}\bigl(1+ \vert U_{n}+v \vert \bigr)^{\gamma}\,dv \\ &\leq \vert a_{n}\varepsilon_{0} \vert ^{2} \bigl[\bigl(1+ \vert U_{n} \vert \bigr)^{\gamma}+\bigl(1+ \vert U_{n}+a_{n}\varepsilon _{0} \vert \bigr)^{\gamma}\bigr] \\ &\leq C \vert a_{n}\varepsilon_{0} \vert ^{2}\bigl[\bigl(1+ \vert U_{n} \vert \bigr)^{\gamma}+ \vert a_{n}\varepsilon_{0} \vert ^{\gamma}\bigr]. \end{aligned}$$

By $\sup_{j} E\varepsilon_{j}^{2}<\infty$ and Chatterji’s inequality (Lin and Bai [35]), we have

3.37 $$ EU_{n}^{2}\leq\sum_{j\neq n,j=1}^{\infty}{a}_{j}^{2}E \varepsilon _{n-j}^{2}\leq\sum_{j=0}^{\infty}a_{j}^{2}. $$

By (3.33)–(3.37) and the Schwartz inequality, we have

3.38 $$\begin{aligned} E(R_{n})&\leq CE\bigl\{ \vert a_{n}\varepsilon_{0} \vert ^{2}+ \vert a_{n}\varepsilon_{0} \vert ^{2+\gamma }+ \vert a_{n}\varepsilon_{0} \vert ^{2} \vert U_{n} \vert ^{\gamma}\bigr\} \\ &\leq C \bigl\{ \vert 1+|a_{n} \vert ^{\gamma}+E\bigl[ \vert \varepsilon_{0} \vert ^{2} \vert U_{n} \vert ^{\gamma}\bigr]\bigr\} \\ &\leq C a_{n}^{2}\bigl\{ \vert 1+|a_{n} \vert ^{\gamma}+\bigl(E \vert U_{n} \vert ^{2} \bigr)^{\gamma/2}\bigr\} \\ &\leq C a_{n}^{2}\Biggl\{ \vert 1+|a_{n} \vert ^{\gamma}+\Biggl(\sum_{j=0}^{\infty}a_{j}^{2}\Biggr)^{\gamma/2}\Biggr\} . \end{aligned}$$

Note that $\sum_{j=0}^{\infty}|a_{j}|<\infty$ implies $\sum_{j=0}^{\infty}a_{j}^{2}<\infty$ and $\sum_{j=0}^{\infty}|a_{j}|^{1+\gamma/2}<\infty$, and by (3.33) and (3.39), we have

3.39 $$ \sum_{i=0}^{\infty}\omega(i)\leq\sum _{n=0}^{\infty}\max \bigl( \vert a_{n} \vert , \vert a_{n} \vert ^{1+\gamma/2}\bigr)< \infty. $$

The general case $k\geq1$ similarly follows. Similar to the proof of (3.39), we easily prove the other results. □

From Propositions 2.1 and 3.2, (A6) and (A7) hold. Hence, we can obtain the following two corollaries from Corollaries 2.1 and 2.2. In order to prove the following two corollaries, we first give some lemmas.

#### Lemma 3.7

(Liptser and Shiryayev [36])

*Let* $\xi=(\xi_{k})_{-\infty < k<\infty}$ *be a strictly stationary sequence on a probability space* $(\Omega,\mathcal{F},P)$, *and* $\mathcal{G}$ *be a* *σ*-*algebra of invariant sets of the sequence* *ξ* *and* $\mathcal{F}_{k}=\sigma(\ldots ,\xi_{k-1},\xi_{k})$. *For a certain* $p\geq2$, *let* $E|\xi_{0}|^{p}<\infty$ *and* $\sum_{k\geq1}\gamma_{k}(p)<\infty$, *where* $\gamma_{k}(p)=\{E|E(\xi _{k}|F_{0})|^{\frac{p}{p-1}}\}^{\frac{p-1}{p}}$. *Then*

$$Z_{n}=\frac{1}{\sqrt {n}}\sum_{k=1}^{n} \xi_{k}\overset{d}{\longrightarrow}Z(\textit{stably}), $$

*where the random variable* *Z* *has the characteristic function* $E\exp (-\frac{1}{2}\lambda^{2}\sigma^{2})$, *and* $\sigma^{2}=E(\xi_{0}^{2}|\mathcal {G})+2\sum_{k\geq1}E(\xi_{0}\xi_{k}|\mathcal{G})$.

#### Corollary 3.5

*Assume that* (A1)–(A5) *hold*, $\varphi(t)=t\varphi '(0)+O(t^{2})$ *and* $m(t)=O(|t|^{\lambda})$ *for some* $\lambda>0$ *as* $t\rightarrow0$, $\Omega(\hat{\beta}_{n})=O_{p}(r_{n})$. *Under the conditions of Proposition *3.2, $E|\psi(e_{k})|^{\frac{p}{p-1}}<\infty,p\geq2$ *and* $\sum_{k=1}^{n}|x_{kn}|<\infty$, *we have*

3.40 $$ n^{-1/2}\hat{\beta}_{n}\overset{d}{\longrightarrow}Z(\textit{stably}), $$

*where the random variable* *Z* *has the characteristic function* $E\exp (-\frac{1}{2}\lambda^{2}\sigma^{2})$, *and* $\sigma^{2}=(\varphi '(0))^{-2}x_{1n}^{T}x_{1n}E(\psi^{2}(e_{1})|\mathcal{G})+2(\varphi ^{\prime}(0))^{-2}x_{1n}^{T}\sum_{k\geq2}x_{kn}E(\psi(e_{1})\psi (e_{k})|\mathcal{G})$.

#### Proof

By Proposition 2.1, Proposition 3.2 and Corollary 2.1, we have

3.41 $$\begin{aligned} n^{-1/2}\hat{\beta_{n}}&=n^{-1/2}\bigl( \varphi'(0)\bigr)^{-1}\sum_{i=1}^{n} \psi(e_{i})x_{in}+O_{p}\bigl(n^{-1/2} \bigl(r_{n}^{\lambda}\log ^{1/2}n+r_{n}\bigr) \bigr) \\ &=n^{-1/2}\bigl(\varphi'(0)\bigr)^{-1}\sum _{i=1}^{n}\psi(e_{i})x_{in}+o_{p}(1). \end{aligned}$$

By $E|\psi(e_{k})|^{\frac{p}{p-1}}<\infty$ and $\sum_{k=1}^{n}|x_{kn}|<\infty$, we have

3.42 $$\begin{aligned} &\gamma_{k}(p)=\bigl\{ E \bigl\vert E\bigl(\psi(e_{k})x_{kn}| \mathcal{F}_{0}\bigr) \bigr\vert ^{\frac{p}{p-1}}\bigr\} ^{\frac{p-1}{p}} \\ &\phantom{\gamma_{k}(p)}\leq\bigl\{ E\bigl[E\bigl( \bigl\vert \psi(e_{k})x_{kn} \bigr\vert ^{\frac{p}{p-1}}|\mathcal{F}_{0}\bigr)\bigr]\bigr\} ^{\frac {p-1}{p}} \\ &\phantom{\gamma_{k}(p)}=\bigl\{ E \bigl\vert \psi(e_{k})x_{kn} \bigr\vert ^{\frac{p}{p-1}}\bigr\} ^{\frac{p-1}{p}}\}\leq C \vert x_{kn} \vert , \end{aligned}$$

3.43 $$\begin{aligned} &\sum_{k\geq1}\gamma_{k}(p)=\sum _{k=1}^{n} \vert x_{kn} \vert < \infty \end{aligned}$$

and

$$\begin{aligned} \sigma^{2}&=\bigl(\varphi'(0) \bigr)^{-2}E\bigl(\bigl(\psi(e_{1})x_{1n} \bigr)^{2}|\mathcal{G}\bigr)+2\bigl(\varphi '(0) \bigr)^{-2}\sum_{k\geq2}E\bigl( \psi(e_{1})x_{1n}\psi(e_{k})x_{kn}| \mathcal {G}\bigr) \\ &=\bigl(\varphi'(0)\bigr)^{-2}x_{1n}^{2}E \bigl(\psi^{2}(e_{1})|\mathcal{G}\bigr)+2\bigl(\varphi '(0)\bigr)^{-2}x_{1n}\sum _{k\geq2}x_{kn}E\bigl(\psi(e_{1}) \psi(e_{k})|\mathcal{G}\bigr). \end{aligned}$$

By Proposition 2.1, Proposition 3.2 and Corollary 2.2, we easily obtain the following result. Here we omit the proof. □

#### Corollary 3.6

*Assume that* (A1)–(A5) *hold*, $\varphi(t)=t\varphi '(0)+O(t^{2})$ *and* $m(t)=O(\sqrt {t})$ *as* $t\rightarrow0$, $\tilde{\Omega}_{n}(\tilde{\beta}_{n})=O_{a.s.}(\tilde{r}_{n})$. *Under the conditions of Proposition *3.2, *we have*

$$ \tilde{\beta}_{n}=O_{a.s}\bigl(n^{-1/2}(\log n)^{3/2}(\log\log n)^{1/{2+\upsilon}}\bigr),\quad \upsilon> 0. $$

## Proofs of the main results

For the proofs of Theorem 2.1 and Theorem 2.2, we need some lemmas as follows.

### Lemma 4.1

(Freedman [21])

*Let* *τ* *be a stopping time*, *and* *K* *a positive real number*. *Suppose that* $P\{|\xi_{i}|\leq K,i\leq\tau\}=1$, *where* $\{\xi_{i}\}$ *are measurable random variables and* $E(\xi_{i}|\mathcal {F}_{i-1})=0$. *Then*, *for all positive real numbers* *a* *and* *b*,

$$\begin{aligned} P\Biggl\{ \sum_{i=1}^{n} \xi_{i} \geq a \textit{ and } T_{n}\leq b,\textit{ for some } n\leq \tau\Biggr\} &\leq\biggl(\biggl(\frac{b}{Ka+b}\biggr)^{Ka+b}e^{Ka} \biggr)^{K^{-2}} \\ &\leq \exp\biggl(-\frac{a^{2}}{2(Ka+b)}\biggr). \end{aligned}$$

### Lemma 4.2

*Let*

4.1 $$ M_{n}(\beta_{n})=\sum_{i=1}^{n} \bigl\{ \psi\bigl(e_{i}-x_{in}^{T} \beta_{n}\bigr)-E\bigl(\psi \bigl(e_{i}-x_{in}^{T} \beta_{n}\bigr)|\mathcal{F}_{i-1}\bigr)\bigr\} x_{in}. $$

*Assume that* (A5) *and* (A6) *hold*. *Then*

4.2 $$ \sup_{ \vert \beta_{n} \vert \leq\delta_{n}} \bigl\vert M_{n}(\beta_{n})-M_{n}(0) \bigr\vert =O_{p}\bigl(\sqrt{\tau _{n}( \delta_{n})}\log n+n^{-3}\bigr). $$

### Proof

Note that $p=\sum_{i=1}^{n} x_{in}^{T}x_{in}\leq(\max_{1\leq i\leq n}|x_{in}|)^{2}n=nr_{n}^{2}$, and $\delta_{n}r_{n}\rightarrow 0$, we have $\delta_{n}=o(n^{1/2})$. For any positive sequence $\mu_{n}\rightarrow\infty$, let

$$\begin{aligned} &\phi_{n}=2\mu_{n}\sqrt{\tau_{n}( \delta_{n})}\log n,\qquad t_{n}=\mu_{n}\sqrt{ \tau_{n}(\delta _{n})}/\log\mu_{n},\qquad u_{n}=t_{n}^{2}, \\ &\eta_{i}(\beta_{n})=\bigl(\psi\bigl(e_{i}-x_{in}^{T} \beta_{n}\bigr)-\psi(e_{i})\bigr)x_{in},\qquad T_{n}= \max_{1\leq i\leq n}\sup_{ \vert \beta_{n} \vert \leq\delta_{n}} \bigl\vert \eta_{i}(\beta _{n}) \bigr\vert \end{aligned}$$

and

$$ U_{n}=\sum_{i=1}^{n}E\bigl\{ \bigl[\psi\bigl(e_{i}+ \vert x_{in} \vert \delta_{n}\bigr)-\psi \bigl(e_{i}- \vert x_{in} \vert \delta_{n}\bigr)\bigr]^{2}|\mathcal{F}_{i-1} \bigr\} \vert x_{in} \vert ^{2}. $$

By the monotonicity of *ψ* and $\delta\geq0$, we have

4.3 $$\begin{aligned} \sup_{ \vert \beta_{n} \vert \leq\delta} \bigl\vert \eta_{i}( \beta_{n}) \bigr\vert &\leq \vert x_{in} \vert \sup _{ \vert \beta_{n} \vert \leq\delta} \bigl\vert \psi\bigl(e_{i}-x_{in}^{T} \beta_{n}\bigr)-\psi(e_{i}) \bigr\vert \\ &\leq \vert x_{in} \vert \max\bigl\{ \psi\bigl(e_{i}- \vert x_{in} \vert \delta\bigr)-\psi(e_{i}),\psi \bigl(e_{i}+ \vert x_{in} \vert \delta\bigr)- \psi(e_{i})\bigr\} \\ &\leq \vert x_{in} \vert \bigl\{ \psi\bigl(e_{i}+ \vert x_{in} \vert \delta\bigr)-\psi\bigl(e_{i}- \vert x_{in} \vert \delta\bigr) \bigr\} . \end{aligned}$$

By (4.3), the $c_{r}$-inequality and (A3), we have

$$\begin{aligned} E\Bigl(\sup_{ \vert \beta_{n} \vert \leq\delta_{n}} \bigl\vert \eta_{i}( \beta_{n}) \bigr\vert ^{2}\Bigr)&\leq E\bigl\{ \vert x_{in} \vert \bigl[\psi\bigl(e_{i}+ \vert x_{in} \vert \delta\bigr)-\psi\bigl(e_{i}- \vert x_{in} \vert \delta\bigr)\bigr]\bigr\} ^{2} \\ &\leq2 \vert x_{in} \vert ^{2}\bigl\{ E\bigl[\psi \bigl(e_{i}+ \vert x_{in} \vert \delta\bigr)- \psi(e_{i})\bigr]^{2}+E\bigl[\psi \bigl(e_{i}- \vert x_{in} \vert \delta\bigr)-\psi(e_{i}) \bigr]^{2}\bigr\} \\ &=2 \vert x_{in} \vert ^{2}\bigl[m^{2}\bigl( \vert x_{in} \vert \delta\bigr)+m^{2}\bigl(- \vert x_{in} \vert \delta\bigr)\bigr]. \end{aligned}$$

Thus

4.4 $$\begin{aligned} E\bigl(T_{n}^{2}\bigr)&=E\Bigl(\max_{1\leq i\leq n} \sup_{ \vert \beta_{n} \vert \leq\delta _{n}} \bigl\vert \eta_{i}( \beta_{n}) \bigr\vert ^{2}\Bigr)\leq\sum _{i=1}^{n}E\Bigl(\sup_{ \vert \beta _{n} \vert \leq\delta_{n}} \bigl\vert \eta_{i}(\beta_{n}) \bigr\vert ^{2} \Bigr) \\ &\leq2\sum_{i=1}^{n} \vert x_{in} \vert ^{2}\bigl[m^{2}\bigl( \vert x_{in} \vert \delta _{n}\bigr)+m^{2}\bigl(- \vert x_{in} \vert \delta_{n}\bigr)\bigr]=2 \tau_{n}(\delta_{n}). \end{aligned}$$

By the Chebyshev inequality,

4.5 $$ P\bigl( \vert T_{n} \vert \geq t_{n}\bigr)\leq E \bigl(T_{n}^{2}\bigr)/t_{n}^{2}\leq 2 \tau_{n}(\delta_{n})/t_{n}^{2}=2 \log^{2}\mu_{n}/\mu_{n}^{2}\rightarrow0. $$

Similarly,

4.6 $$ P\bigl( \vert U_{n} \vert \geq t_{n}\bigr)\leq E(U_{n})/u_{n}=O\bigl((\log\mu_{n}/ \mu_{n})^{2}\bigr)\rightarrow0. $$

Let $x_{in}=(x_{i1n},\ldots,x_{ipn})^{T}=(x_{i1},\ldots ,x_{ip})^{T},D_{x}(i)=(2\times1_{x_{i1}\geq0}-1,\ldots,2\times1_{x_{ip}\geq 0}-1)\in\Pi_{p},\Pi_{p}=\{-1,1\}^{p}$. For $d\in\Pi_{p},j=1,2,\ldots,p$, define

4.7 $$ M_{n,j,d}(\beta_{n})=\sum_{i=1}^{n} \bigl[\psi\bigl(e_{i}-x_{in}^{T} \beta_{n}\bigr)-E\bigl(\psi \bigl(e_{i}-x_{in}^{T} \beta_{n}\bigr)|F_{i-1}\bigr)\bigr]x_{ij}1_{D_{x}(i)=d}. $$

Since $M_{n}(\beta_{n})=\sum_{d\in\Pi_{p}}(M_{n,1,d}(\beta_{n}),\ldots ,M_{n,p,d}(\beta_{n}))^{T}$, it suffices to prove that Lemma 4.2 holds with $M_{n}(\beta_{n})$ replaced by $(M_{n,j,d}(\beta_{n})$.

Let $|\beta_{n}|\leq\delta_{n},\eta_{i,j,d}(\beta_{n})=(\psi(e_{i}-x_{in}^{T}\beta _{n})-\psi(e_{i}))x_{ij}1_{D_{x}(i)=d}$ and

4.8 $$ B_{n}(\beta_{n})=\sum_{i=1}^{n}E \bigl(\eta_{i,j,d}(\beta_{n})1_{ \vert \eta _{i,j,d}(\beta_{n}) \vert >t_{n}}| \mathcal{F}_{i-1}\bigr). $$

Note that

4.9 $$ \frac{u_{n}}{t_{n}\phi_{n}}=\frac{t_{n}}{\phi_{n}}=\frac{\mu_{n}\sqrt{\tau_{n}(\delta _{n})}/\log\mu_{n}}{2\mu_{n}\sqrt{\tau_{n}(\delta_{n})}\log n}=\frac{1}{2\log n\log\mu_{n}} \rightarrow0. $$

By (4.9), for large enough *n*, we have

4.10 $$\begin{aligned} P\bigl( \bigl\vert B_{n}(\beta_{n}) \bigr\vert \geq \phi_{n},U_{n}\leq u_{n}\bigr)&=P\Biggl( \Biggl\vert \sum_{i=1}^{n}E\bigl(\eta _{i,j,d}(\beta_{n})1_{ \vert \eta_{i,j,d}(\beta_{n}) \vert >t_{n}}|\mathcal{F}_{i-1} \bigr) \Biggr\vert \geq \phi_{n},U_{n}\leq u_{n} \Biggr) \\ &\leq P\Biggl(t_{n}^{-1}\sum_{i=1}^{n}E \bigl(\eta_{i,j,d}(\beta_{n})1_{ \vert \eta _{i,j,d}(\beta_{n}) \vert >t_{n}}| \mathcal{F}_{i-1}\bigr)\geq\phi_{n},U_{n}\leq u_{n}\Biggr) \\ &\leq P\bigl(t_{n}^{-1}U_{n}\geq \phi_{n},U_{n}\leq u_{n}\bigr)=P(t_{n} \phi_{n}\leq U_{n}\leq u_{n})=0. \end{aligned}$$

Let the projections $\mathcal{P}_{k}(\cdot)=E(\cdot|\mathcal{F}_{k})-E(\cdot |\mathcal{F}_{k-1})$. Since

4.11 $$\begin{aligned} &E\bigl\{ \mathcal{P}_{i}\bigl(\eta_{i,j,d}( \beta_{n})1_{ \vert \eta_{i,j,d}(\beta_{n}) \vert \leq t_{n}}\bigr)|\mathcal{F}_{i-1}\bigr\} \\ &\quad=E\bigl\{ \bigl[E\bigl(\eta_{i,j,d}(\beta_{n})1_{ \vert \eta_{i,j,d}(\beta_{n}) \vert \leq t_{n}}| \mathcal{F}_{i}\bigr)-E\bigl(\eta_{i,j,d}( \beta_{n})1_{ \vert \eta_{i,j,d}(\beta _{n}) \vert \leq t_{n}}|\mathcal{F}_{i-1}\bigr)\bigr]| \mathcal{F}_{i-1}\bigr\} \\ &\quad=E\bigl(\eta_{i,j,d}(\beta_{n})1_{ \vert \eta_{i,j,d}(\beta_{n}) \vert \leq t_{n}}|\mathcal {F}_{i-1}\bigr)-E\bigl(\eta_{i,j,d}(\beta_{n})1_{ \vert \eta_{i,j,d}(\beta_{n}) \vert \leq t_{n}}| \mathcal{F}_{i-1}\bigr)=0. \end{aligned}$$

Note that $\{\mathcal{P}_{i}(\eta_{i,j,d}(\beta_{n})1_{|\eta_{i,j,d}(\beta _{n})|\leq t_{n}})\}$ are bound martingale differences. By Lemma 4.1 and (4.10), for $|\beta_{n}|\leq t_{n}$, we have

4.12 $$\begin{aligned} &P\bigl\{ \bigl\vert M_{n,j,d}(\beta_{n})-M_{n,j,d}(0) \bigr\vert \geq2\phi_{n},T_{n}\leq t_{n},U_{n} \leq u_{n}\bigr\} \\ &\quad\leq P\Biggl\{ \Biggl\vert \sum_{i=1}^{n} \mathcal{P}_{i}\bigl(\eta_{i,j,d}(\beta_{n})1_{ \vert \eta _{i,j,d}(\beta_{n}) \vert \leq t_{n}} \bigr) \Biggr\vert \geq\phi_{n},T_{n}\leq t_{n},U_{n}\leq u_{n}\Biggr\} \\ &\qquad{}+P\Biggl\{ \Biggl\vert \sum_{i=1}^{n} \mathcal{P}_{i}\bigl(\eta_{i,j,d}(\beta_{n})1_{ \vert \eta _{i,j,d}(\beta_{n}) \vert > t_{n}} \bigr) \Biggr\vert \geq\phi_{n},T_{n}\leq t_{n},U_{n}\leq u_{n}\Biggr\} \\ &\quad\leq C\exp\biggl(-\frac{\phi_{n}^{2}}{4t_{n}\phi_{n}+2u_{n}}\biggr)+P\bigl( \bigl\vert B_{n}(\beta_{n}) \bigr\vert \geq\phi _{n},U_{n} \leq u_{n}\bigr) \\ &\quad=O\biggl(\exp\biggl(-\frac{\phi_{n}^{2}}{4t_{n}\phi_{n}+2u_{n}}\biggr)\biggr). \end{aligned}$$

Let $l=n^{8}$ and $K_{l}=\{(k_{1}/l,\ldots,k_{p}/l):k_{i}\in Z,|k_{i}|\leq n^{9}\}$. Then $\# K_{l}=(2n^{9}+1)^{p}$, where the symbol # denotes the number of elements of the set $K_{l}$. It is easy to show

4.13 $$ t_{n}\phi_{n}\log n=o\bigl(\phi_{n}^{2} \bigr) \quad\text{and} \quad u_{n}\log n=o\bigl(\phi_{n}^{2}\bigr). $$

By (4.12) and (4.13), for $\forall\varsigma>1$, we have

4.14 $$\begin{aligned} &P\Bigl\{ \sup_{\beta_{n}\in K_{l}} \bigl\vert M_{n,j,d}( \beta_{n})-M_{n,j,d}(0) \bigr\vert \geq 2\phi_{n},T_{n} \leq t_{n},U_{n}\leq u_{n}\Bigr\} \\ &\quad\leq\sum_{\# K_{l}}P\bigl\{ \bigl\vert M_{n,j,d}(\beta_{n})-M_{n,j,d}(0) \bigr\vert \geq2 \phi _{n},T_{n}\leq t_{n},U_{n}\leq u_{n}\bigr\} \\ &\quad\leq Cn^{9p}\exp\biggl(-\frac{\phi_{n}^{2}}{4t_{n}\phi_{n}+2u_{n}}\biggr)=Cn^{9p} \exp\biggl(-\frac {\log n}{4t_{n}\phi_{n}\log n/\phi_{n}^{2}+2u_{n}\log n/\phi_{n}^{2}}\biggr) \\ &\quad=Cn^{9p}\exp\biggl(-\frac{\log n}{o(1)}\biggr)=o\bigl(n^{-\varsigma p} \bigr). \end{aligned}$$

By (4.5), (4.6) and (4.14), we have

4.15 $$ P\Bigl\{ \sup_{\beta_{n}\in K_{l}} \bigl\vert M_{n,j,d}( \beta_{n})-M_{n,j,d}(0) \bigr\vert \geq 2\phi_{n} \Bigr\} \rightarrow0,\quad n\rightarrow\infty. $$

For *a*, let $\langle a\rangle_{l,-1}=\lceil a\rceil_{l}=\lceil al\rceil /l$ and $\langle a\rangle_{l,1}=\lfloor a\rfloor_{l}=\lfloor al\rfloor /l$. For a vector $\beta_{n}=(\beta_{1n},\ldots,\beta_{pn})^{T}$, let $\langle\beta_{n}\rangle_{l,d}=(\langle\beta_{1n}\rangle_{l,d_{1}},\ldots ,\langle\beta_{pn}\rangle_{l,d_{p}})$.

By (A5), for $|s|,|t|\leq r_{n}\delta_{n}$ and large *n*, we have

$$\bigl\vert E\bigl\{ \bigl[\psi(e_{i}-t)-\psi(e_{i}-s) \bigr]|\mathcal{F}_{i-1}\bigr\} \bigr\vert \leq L_{i-1} \vert s-t \vert . $$

Let $V_{n}=\sum_{i=1}^{n}L_{i-1}$. By condition (A5), the Markov inequality and $L_{i}\in L^{1}$, we have

4.16 $$ P\bigl(V_{n}\geq n^{4}\bigr)\leq EV_{n}/n^{4}= \sum_{i=1}^{n}EL_{i-1}/n^{4} \leq Cn^{-3}. $$

Note that $|\beta_{n}-\langle\beta_{n}\rangle_{l,d}|\leq Cl^{-1}$, which implies $\max_{1\leq i\leq n}|x_{in}^{T}(\beta_{n}-\langle\beta _{n}\rangle_{l,d})|=o(l^{-1})$. Thus

4.17 $$\begin{aligned} &\sup_{ \vert \beta_{n} \vert \leq\delta_{n}} \Biggl\vert \sum_{i=1}^{n}E \bigl\{ \bigl[\eta _{i}\bigl(\langle\beta_{n} \rangle_{l,d}\bigr)-\eta_{i}(\beta_{n})\bigr]| \mathcal{F}_{i-1}\bigr\} x_{in} \Biggr\vert \\ &\quad\leq\sup_{ \vert \beta_{n} \vert \leq\delta_{n}}\sum_{i=1}^{n} \bigl\vert E\bigl(\bigl(\psi \bigl(e_{i}-x_{in}^{T} \langle\beta_{n}\rangle_{l,d}\bigr)-\psi(e_{i}) \bigr)-\bigl(\psi \bigl(e_{i}-x_{in}^{T} \beta_{n}\bigr)-\psi(e_{i})\bigr)|\mathcal{F}_{i-1} \bigr)x_{in} \bigr\vert \\ &\quad=\sup_{ \vert \beta_{n} \vert \leq\delta_{n}}\sum_{i=1}^{n} \bigl\vert E\bigl(\psi \bigl(e_{i}-x_{in}^{T} \langle\beta_{n}\rangle_{l,d}\bigr)-\psi\bigl(e_{i}-x_{in}^{T} \beta _{n}\bigr)|\mathcal{F}_{i-1}\bigr)x_{in} \bigr\vert \\ &\quad\leq\sup_{ \vert \beta_{n} \vert \leq\delta_{n}}\sum_{i=1}^{n} \vert x_{in} \vert L_{i-1} \bigl\vert x_{in}^{T}\bigl(\langle\beta_{n} \rangle_{l,d}-\beta _{n}\bigr) \bigr\vert \leq Cl^{-1}V_{n}. \end{aligned}$$

Without loss of generality, assume that $j=1$ in the following proof.

Let $d=(1,-1,1,\ldots,1)$. Then $\langle\beta_{n}\rangle_{l,d}=(\lfloor \beta_{1n}\rfloor_{l},\lceil\beta_{2n}\rceil_{l},\lfloor\beta_{3n}\rfloor _{l},\ldots,\lfloor\beta_{pn}\rfloor_{l})$ and $\langle\beta_{n}\rangle _{l,-d}=(\lceil\beta_{1n}\rceil_{l},\lfloor\beta_{2n}\rfloor_{l},\lceil \beta_{3n}\rceil_{l},\ldots,\lceil\beta_{pn}\rceil_{l})$. Since *ψ* is nondecreasing,

$$\eta_{i,1,d}\bigl(\langle\beta_{n}\rangle_{l,-d} \bigr)\leq\eta_{i,1,d}(\beta_{n})\leq \eta_{i,1,d}\bigl( \langle\beta_{n}\rangle_{l,d}\bigr). $$

Note that

$$\begin{aligned} &\eta_{i,1,d}\bigl(\langle\beta_{n}\rangle_{l,-d} \bigr)-E\bigl[\eta_{i,1,d}\bigl(\langle \beta_{n} \rangle_{l,-d}\bigr)|\mathcal{F}_{i-1}\bigr]+E\bigl[ \eta_{i,1,d}\bigl(\langle\beta _{n}\rangle_{l,-d} \bigr)|\mathcal{F}_{i-1}\bigr]\\ &\qquad{}-E\bigl[\eta_{i,1,d}( \beta_{n})|\mathcal {F}_{i-1}\bigr] \\ &\quad\leq\eta_{i,1,d}(\beta_{n})-E\bigl[\eta_{i,1,d}( \beta_{n})|\mathcal{F}_{i-1}\bigr] \\ &\quad\leq\eta_{i,1,d}\bigl(\langle\beta_{n}\rangle_{l,d} \bigr)-E\bigl[\eta_{i,1,d}\bigl(\langle \beta_{n} \rangle_{l,d}\bigr)|\mathcal{F}_{i-1}\bigr]+E\bigl[ \eta_{i,1,d}\bigl(\langle\beta _{n}\rangle_{l,d} \bigr)|\mathcal{F}_{i-1}\bigr]\\ &\qquad{}-E\bigl[\eta_{i,1,d}( \beta_{n})|\mathcal{F}_{i-1}\bigr]. \end{aligned}$$

Namely

$$\begin{aligned} &\sum_{i=1}^{n}\bigl\{ \eta_{i,1,d} \bigl(\langle\beta_{n}\rangle_{l,-d}\bigr)-E\bigl[\eta _{i,1,d}\bigl(\langle\beta_{n}\rangle_{l,-d}\bigr)| \mathcal{F}_{i-1}\bigr]\\ &\qquad{}+E\bigl[\bigl(\eta _{i,1,d}\bigl(\langle \beta_{n}\rangle_{l,-d}\bigr)-\eta_{i,1,d}( \beta_{n})\bigr)|\mathcal {F}_{i-1}\bigr]\bigr\} x_{i1}1_{D_{x}(i)=d} \\ &\quad\leq\sum_{i=1}^{n}\bigl\{ \eta_{i,1,d}(\beta_{n})-E\bigl[\eta_{i,1,d}(\beta _{n})|\mathcal{F}_{i-1}\bigr]\bigr\} x_{i1}1_{D_{x}(i)=d} \\ &\quad\leq\sum_{i=1}^{n}\bigl\{ \eta_{i,1,d}\bigl(\langle\beta_{n}\rangle _{l,d} \bigr)-E\bigl[\eta_{i,1,d}\bigl(\langle\beta_{n} \rangle_{l,d}\bigr)|\mathcal {F}_{i-1}\bigr]\\ &\qquad{}+E\bigl[\bigl( \eta_{i,1,d}\bigl(\langle\beta_{n}\rangle_{l,d}\bigr)- \eta _{i,1,d}(\beta_{n})\bigr)|\mathcal{F}_{i-1}\bigr] \bigr\} x_{i1}1_{D_{x}(i)=d}. \end{aligned}$$

Therefore

4.18 $$\begin{aligned} &M_{n,1,d}\bigl(\langle\beta_{n}\rangle_{l,-d} \bigr)-M_{n,1,d}(0)+\sum_{i=1}^{n}E \bigl\{ \bigl[\eta_{i,1,d}\bigl(\langle\beta_{n} \rangle_{l,-d}\bigr)-\eta _{i,1,d}(\beta_{n})\bigr]| \mathcal{F}_{i-1}\bigr\} x_{i1}1_{D_{x}(i)=d} \\ &\quad\leq M_{n,1,d}(\beta_{n})-M_{n,1,d}(0) \\ &\quad\leq M_{n,1,d}\bigl(\langle\beta_{n}\rangle_{l,d} \bigr)-M_{n,1,d}(0) \\ &\qquad{}+\sum_{i=1}^{n}E \bigl\{ \bigl[\eta_{i,1,d}\bigl(\langle\beta_{n} \rangle_{l,d}\bigr)-\eta _{i,1,d}(\beta_{n})\bigr]| \mathcal{F}_{i-1}\bigr\} x_{i1}1_{D_{x}(i)=d}. \end{aligned}$$

By (4.17) and (4.18), we have

4.19 $$\begin{aligned} M_{n,1,d}\bigl(\langle\beta_{n}\rangle_{l,-d} \bigr)-M_{n,1,d}(0)-Cl^{-1}V_{n}&\leq M_{n,1,d}(\beta_{n})-M_{n,1,d}(0) \\ &\leq M_{n,1,d}\bigl(\langle\beta_{n}\rangle_{l,d} \bigr)-M_{n,1,d}(0)+Cl^{-1}V_{n}. \end{aligned}$$

Note that $l^{-1}V_{n}=O_{p}(n^{-8}n^{4})=O_{p}(n^{-4})$, (4.2) immediately follows from (4.15) and (4.19). □

### Lemma 4.3

*Assume that the processes* $X_{t}=g(\mathcal{F}_{t})\in L^{2}$. *Let* $g_{n}(\mathcal{F}_{0})=E(g(\mathcal{F}_{n})|\mathcal{F}_{0}),n\geq 0$. *Then*

4.20 $$\begin{aligned} \begin{aligned} &\bigl\Vert g_{n}(\mathcal{F}_{0})-g_{n}\bigl( \mathcal{F}_{0}^{*}\bigr) \bigr\Vert \leq \bigl\Vert g(\mathcal {F}_{n})-g\bigl(\mathcal{F}_{n}^{*}\bigr) \bigr\Vert ,\\ &\Vert \mathcal{P}_{0}X_{n} \Vert \leq \bigl\Vert g_{n}(\mathcal {F}_{0})-g_{n}\bigl( \mathcal{F}_{0}^{*}\bigr) \bigr\Vert +R, \end{aligned} \end{aligned}$$

*where* $R= \Vert E[g_{n}(\mathcal{F}_{0}^{*})|\mathcal{F}_{-1}]-E[g_{n}(\mathcal {F}_{0}^{*})|\mathcal{F}_{0}] \Vert $.

### Proof

Since

$$\begin{aligned} E\bigl\{ \bigl[g(\mathcal{F}_{n})-g\bigl(\mathcal{F}_{n}^{*} \bigr)\bigr]|\bigl(\mathcal{F}_{-1},\varepsilon '_{0}, \varepsilon_{0}\bigr)\bigr\} &=E\bigl[g(\mathcal{F}_{n})|( \mathcal{F}_{-1},\varepsilon _{0})\bigr]-E\bigl[g\bigl( \mathcal{F}_{n}^{*}\bigr)|\bigl(\mathcal{F}_{-1}, \varepsilon'_{0}\bigr)\bigr] \\ &=g_{n}(\mathcal{F}_{0})-g_{n}\bigl( \mathcal{F}_{0}^{*}\bigr), \end{aligned}$$

we have

4.21 $$ E \bigl\vert E\bigl\{ \bigl[g(\mathcal{F}_{n})-g\bigl( \mathcal{F}_{n}^{*}\bigr)\bigr]|\bigl(\mathcal{F}_{-1}, \varepsilon '_{0},\varepsilon_{0}\bigr)\bigr\} \bigr\vert ^{2}=E \bigl\vert g_{n}( \mathcal{F}_{0})-g_{n}\bigl(\mathcal{F}_{0}^{*} \bigr) \bigr\vert ^{2}. $$

By the Jensen inequality, we have

4.22 $$\begin{aligned} E \bigl\vert E\bigl\{ \bigl[g(\mathcal{F}_{n})-g\bigl( \mathcal{F}_{n}^{*}\bigr)\bigr]|\bigl(\mathcal{F}_{-1}, \varepsilon '_{0},\varepsilon_{0}\bigr)\bigr\} \bigr\vert ^{2}&\leq E\bigl\{ E\bigl[ \bigl\vert g( \mathcal{F}_{n})-g\bigl(\mathcal {F}_{n}^{*}\bigr) \bigr\vert ^{2}|\bigl(\mathcal{F}_{-1},\varepsilon'_{0}, \varepsilon_{0}\bigr)\bigr]\bigr\} \\ &=E \bigl\vert g(\mathcal{F}_{n})-g\bigl(\mathcal{F}_{n}^{*} \bigr) \bigr\vert ^{2}. \end{aligned}$$

By (4.21) and (4.22), we have

$$E \bigl\vert g_{n}(\mathcal{F}_{0})-g_{n} \bigl(\mathcal{F}_{0}^{*}\bigr) \bigr\vert ^{2}\leq E \bigl\vert g(\mathcal {F}_{n})-g\bigl(\mathcal{F}_{n}^{*}\bigr) \bigr\vert ^{2}. $$

That is,

4.23 $$ \bigl\Vert g_{n}(\mathcal{F}_{0})-g_{n}\bigl( \mathcal{F}_{0}^{*}\bigr) \bigr\Vert \leq \bigl\Vert g(\mathcal {F}_{n})-g\bigl(\mathcal{F}_{n}^{*}\bigr) \bigr\Vert . $$

Note that

4.24 $$ E\bigl[g_{n}(\mathcal{F}_{0})|\mathcal{F}_{-1} \bigr]=E\bigl[E\bigl(g(\mathcal{F}_{n})|\mathcal {F}_{0} \bigr)|\mathcal{F}_{-1}\bigr]=E\bigl(g_{n}\bigl( \mathcal{F}_{0}^{*}\bigr)|\mathcal{F}_{-1}\bigr) $$

and

4.25 $$ E\bigl[g_{n}(\mathcal{F}_{0})|\mathcal{F}_{-1} \bigr]=E\bigl[g_{n}\bigl(\mathcal{F}_{0}^{*}\bigr)|\mathcal {F}_{0}\bigr]+E\bigl[g_{n}\bigl(\mathcal{F}_{0}^{*} \bigr)|\mathcal{F}_{-1}\bigr]-E\bigl[g_{n}\bigl(\mathcal {F}_{0}^{*}\bigr)|\mathcal{F}_{0}\bigr]. $$

By (4.24), (4.25) and the Jensen inequality, we have

4.26 $$\begin{aligned} \Vert \mathcal{P}_{0}X_{n} \Vert &= \bigl\Vert E \bigl(g(\mathcal{F}_{n})|\mathcal{F}_{0}\bigr)-E\bigl(g( \mathcal {F}_{n})|\mathcal{F}_{-1}\bigr) \bigr\Vert \\ &= \bigl\Vert E\bigl[g_{n}(\mathcal{F}_{0})| \mathcal{F}_{0}\bigr]-E\bigl[g_{n}(\mathcal{F}_{0})| \mathcal {F}_{-1}\bigr] \bigr\Vert \\ &= \bigl\Vert E\bigl[g_{n}(\mathcal{F}_{0})| \mathcal{F}_{0}\bigr]-E\bigl[g_{n}\bigl( \mathcal{F}_{0}^{*}\bigr)|\mathcal {F}_{0}\bigr]-E \bigl[g_{n}\bigl(\mathcal{F}_{0}^{*}\bigr)| \mathcal{F}_{-1}\bigr]+E\bigl[g_{n}\bigl(\mathcal {F}_{0}^{*}\bigr)|\mathcal{F}_{0}\bigr] \bigr\Vert \\ &\leq \bigl\Vert E\bigl[g_{n}(\mathcal{F}_{0})| \mathcal{F}_{0}\bigr]-E\bigl[g_{n}\bigl(\mathcal {F}_{0}^{*}\bigr)|\mathcal{F}_{0}\bigr] \bigr\Vert + \bigl\Vert E\bigl[g_{n}\bigl(\mathcal{F}_{0}^{*}\bigr)| \mathcal {F}_{-1}\bigr]-E\bigl[g_{n}\bigl( \mathcal{F}_{0}^{*}\bigr)|\mathcal{F}_{0}\bigr] \bigr\Vert \\ &\leq \bigl\Vert g_{n}(\mathcal{F}_{0})-g_{n} \bigl(\mathcal{F}_{0}^{*}\bigr) \bigr\Vert +R. \end{aligned}$$

 □

### Remark 4

If $\{\varepsilon_{i}\}$ i.i.d., then $R=0$. In this case, the above lemma becomes Theorem 1 of Wu [48].

### Lemma 4.4

*Let* $\{\delta_{n},n\in N\}$ *be a sequence of positive numbers such that* $\delta_{n}\rightarrow\infty$ *and* $\delta_{n} r_{n}\rightarrow0$. *If* (A6)–(A7) *hold*, *then*

4.27 $$ \Bigl\Vert \sup_{ \vert \beta_{n} \vert \leq\delta_{n}} \bigl\vert N_{n}( \beta_{n})-N_{n}(0) \bigr\vert \Bigr\Vert =O \Biggl(\sqrt {\sum_{i=1}^{n} \vert x_{in} \vert ^{4}}\delta_{n} \Biggr), $$

*where*

$$N_{n}(\beta_{n})=\sum_{i=1}^{n} \bigl\{ \psi_{i}\bigl(-x_{in}^{T} \beta_{n};\mathcal {F}_{i-1}\bigr)-\varphi \bigl(-x_{in}^{T}\beta_{n}\bigr)\bigr\} x_{in}. $$

### Proof

Let $I=\{n_{1},\ldots,n_{q}\}\in\{1,2,\ldots,p\}$ be a nonempty set and $1\leq n_{1}<\cdots<n_{q}$, and $u_{I}=(u_{1}1_{1\in I},\ldots ,u_{p}1_{p\in I})$, with vector $u=(u_{1},\ldots,u_{p})$. Write

$$\begin{aligned} &\int_{0}^{\beta_{n,I}}\frac{\partial^{q}N_{n}(u_{I})}{\partial u_{I}}\,du_{I}\\ &\quad= \int _{0}^{\beta_{n,m_{1}}}\cdots \int_{0}^{\beta_{n,m_{q}}}\frac{\partial ^{q}N_{n}(u_{I})}{\partial u_{m_{1}}\cdots\partial u_{m_{q}}}\,du_{m_{1}} \cdots du_{m_{q}},w_{i}=x_{in}x_{im_{1}} \cdots x_{im_{q}}. \end{aligned}$$

In the following, we will prove that

4.28 $$ \biggl\vert \frac{\partial^{q}N_{n}(u_{I})}{\partial u_{I}} \biggr\vert = \Biggl\vert \sum _{i=1}^{n}\bigl\{ \psi _{i}^{(q)} \bigl(-x_{in}^{T} u_{I};\mathcal{F}_{i-1} \bigr)-\varphi^{(q)}\bigl(-x_{in}^{T}u_{I} \bigr)\bigr\} w_{i} \Biggr\vert =O \Biggl(\sqrt{\sum _{i=1}^{n} \vert x_{in} \vert ^{2+2q}} \Biggr) $$

uniformly over $|u|\leq p\delta_{n}$.

In fact, let

$$T_{n}=\sum_{i=1}^{n}\bigl\{ \psi_{i}^{(q)}\bigl(-x_{in}^{T} u_{I};\mathcal {F}_{i-1}\bigr)-\varphi^{(q)} \bigl(-x_{in}^{T}u_{I}\bigr)\bigr\} w_{i} $$

and

$$J_{k}=\sum_{i=1}^{n} \mathcal{P}_{i-k}\bigl\{ \psi_{i}^{(q)} \bigl(-x_{in}^{T} u_{I};\mathcal{F}_{i-1} \bigr)-\varphi^{(q)}\bigl(-x_{in}^{T}u_{I} \bigr)\bigr\} w_{i}. $$

Then $T_{n}=\sum_{k=0}^{\infty}J_{k}$, and $J_{k}$ are martingale differences. By the orthogonality of martingale differences and the stationarity of $\{e_{i}\}$, and Lemma 4.3, we have

4.29 $$\begin{aligned} \Vert J_{k} \Vert ^{2}&=\sum _{i=1}^{n} \bigl\Vert \mathcal{P}_{i-k} \bigl\{ \psi _{i}^{(q)}\bigl(-x_{in}^{T} u_{I};\mathcal{F}_{i-1}\bigr)-\varphi^{(q)} \bigl(-x_{in}^{T}u_{I}\bigr)\bigr\} w_{i} \bigr\Vert ^{2} \\ &=\sum_{i=1}^{n} \vert w_{i} \vert ^{2} \bigl\Vert \mathcal{P}_{0}\bigl\{ \psi_{k}^{(q)}\bigl(-x_{kn}^{T} u_{I};\mathcal{F}_{k-1}\bigr)-\varphi^{(q)} \bigl(-x_{kn}^{T}u_{I}\bigr)\bigr\} \bigr\Vert ^{2}. \end{aligned}$$

By Lemma 4.3, $\psi_{i}(\cdot;\mathcal{F}_{i-1})\in C^{l},l\geq0$ and the $c_{r}$-inequality, for $k\geq0$, we have

4.30 $$\begin{aligned} & \bigl\Vert \mathcal{P}_{0}\bigl\{ \psi_{k}^{(q)} \bigl(-x_{kn}^{T} u_{I};\mathcal{F}_{k-1} \bigr)-\varphi ^{(q)}\bigl(-x_{kn}^{T}u_{I} \bigr)\bigr\} \bigr\Vert ^{2} \\ &\quad\leq \bigl\Vert E\bigl\{ \bigl[\psi_{k}^{(q)} \bigl(-x_{kn}^{T} u_{I};\mathcal{F}_{k-1} \bigr)-\varphi ^{(q)}\bigl(-x_{kn}^{T}u_{I} \bigr)\bigr]|\mathcal{F}_{0}\bigr\} \\ &\qquad{}-E\bigl\{ \bigl[ \psi_{k}^{(q)}\bigl(-x_{kn}^{T} u_{I};\mathcal{F}_{k-1}\bigr)-\varphi ^{(q)} \bigl(-x_{kn}^{T}u_{I}\bigr)\bigr]| \mathcal{F}_{0}^{*}\bigr\} \bigr\Vert ^{2}+R_{k}^{2} \\ &\quad\leq2 \bigl\Vert E\bigl\{ \psi_{k}^{(q)} \bigl(-x_{kn}^{T} u_{I};\mathcal{F}_{k-1} \bigr)|\mathcal{F}_{0}\bigr\} -E\bigl\{ \psi_{k}^{(q)} \bigl(-x_{kn}^{T} u_{I};\mathcal{F}_{k-1}^{*} \bigr)|\mathcal{F}_{0}^{*}\bigr\} \bigr\Vert ^{2} \\ &\qquad{}+2 \bigl\Vert E\bigl\{ \varphi^{(q)}\bigl(-x_{kn}^{T} u_{I}\bigr)|\mathcal{F}_{0}\bigr\} -E\bigl\{ \varphi ^{(q)}\bigl(-x_{kn}^{T} u_{I}\bigr)| \mathcal{F}_{0}^{*}\bigr\} \bigr\Vert ^{2}+R_{k}^{2} \\ &\quad\leq2 \bigl\Vert E\bigl\{ \psi_{k}^{(q)} \bigl(-x_{kn}^{T} u_{I};\mathcal{F}_{k-1} \bigr)|\mathcal{F}_{0}\bigr\} -E\bigl\{ \psi_{k}^{(q)} \bigl(-x_{kn}^{T} u_{I};\mathcal{F}_{k-1}^{*} \bigr)|\mathcal{F}_{0}^{*}\bigr\} \bigr\Vert ^{2} \\ &\qquad{}+2 \bigl\vert E\psi^{(q)}\bigl(e_{k}-x_{kn}^{T} u_{I}\bigr)-E\psi^{(q)}\bigl(e_{k}^{*}-x_{kn}^{T} u_{I}\bigr) \bigr\vert ^{2}+R_{k}^{2}, \end{aligned}$$

where

$$\begin{aligned} R_{k}^{2}={}& \bigl\Vert E\bigl\{ \bigl[\psi_{k}^{(q)} \bigl(-x_{kn}^{T} u_{I};\mathcal{F}_{k-1}^{*} \bigr)-\varphi ^{(q)}\bigl(-x_{kn}^{T}u_{I} \bigr)\bigr]|\mathcal{F}_{-1}\bigr\} \\ &{}-E\bigl\{ \bigl[\psi_{k}^{(q)} \bigl(-x_{kn}^{T} u_{I};\mathcal{F}_{k-1}^{*} \bigr)-\varphi^{(q)}\bigl(-x_{kn}^{T}u_{I} \bigr)\bigr]|\mathcal{F}_{0}\bigr\} \bigr\Vert ^{2}. \end{aligned}$$

Note that $E\psi^{(q)}(e_{i}+\delta)=\frac{d^{q}E\psi (e_{i}+t)}{dt^{q}}|_{t=\delta}$, we have

4.31 $$\begin{aligned} R_{k}^{2}\leq{}& \bigl\Vert E\bigl[\psi_{k}^{(q)} \bigl(-x_{kn}^{T} u_{I};\mathcal{F}_{k-1}^{*} \bigr)|\mathcal {F}_{-1}\bigr]-E\bigl[\psi_{k}^{(q)} \bigl(-x_{kn}^{T} u_{I};\mathcal{F}_{k-1}^{*} \bigr)|\mathcal {F}_{0}\bigr] \bigr\Vert ^{2} \\ &{}+ \bigl\Vert E\bigl[\varphi^{(q)}\bigl(-x_{kn}^{T} u_{I}\bigr)|F_{-1}\bigr]-E\bigl[\varphi^{(q)} \bigl(-x_{kn}^{T} u_{I}\bigr)| \mathcal{F}_{0}\bigr] \bigr\Vert ^{2} \\ ={}& \bigl\Vert E\bigl[\psi_{k}^{(q)}\bigl(-x_{kn}^{T} u_{I};\mathcal{F}_{k-1}^{*}\bigr)|\mathcal {F}_{-1} \bigr]-E\bigl[\psi_{k}^{(q)}\bigl(-x_{kn}^{T} u_{I};\mathcal{F}_{k-1}^{*}\bigr)|\mathcal {F}_{0}\bigr] \bigr\Vert ^{2} \\ &{}+ \bigl\Vert E\psi_{k}^{(q)}\bigl(e_{k}-x_{kn}^{T} u_{I}\bigr)-E\psi_{k}^{(q)}\bigl(e_{k}-x_{kn}^{T} u_{I}\bigr) \bigr\Vert ^{2} \\ ={}& \bigl\Vert E\bigl[\psi_{k}^{(q)}\bigl(-x_{kn}^{T} u_{I};\mathcal{F}_{k-1}^{*}\bigr)|\mathcal {F}_{-1} \bigr]-E\bigl[\psi_{k}^{(q)}\bigl(-x_{kn}^{T} u_{I};\mathcal{F}_{k-1}^{*}\bigr)|\mathcal {F}_{0}\bigr] \bigr\Vert ^{2}. \end{aligned}$$

By the conditions (A6), (A7) and (4.29)–(4.31), we have

$$\begin{aligned} \Vert T_{n} \Vert &=\sqrt{\sum _{i=1}^{n} \vert w_{i} \vert ^{2}}\sum_{k=0}^{\infty} \Vert J_{k} \Vert =O \Biggl(\sqrt{\sum _{i=1}^{n} \vert w_{i} \vert ^{2}} \Biggr)\\ &=O \Biggl(\sqrt{\sum _{i=1}^{n} \vert x_{in} \vert ^{2+2q}} \Biggr). \end{aligned}$$

Let $|u|\leq p\delta_{n}$. By $\max_{1\leq i\leq n}|x_{in}u|\leq p\delta_{n} r_{n}\rightarrow0$. Note that $\delta_{n}\rightarrow\infty$ and $\delta_{n} r_{n}\rightarrow0$. By (4.28), we have

4.32 $$\begin{aligned} \biggl\Vert \sup_{ \vert \beta_{n} \vert \leq\delta_{n}} \int_{0}^{\beta_{n,I}} \biggl\vert \frac{\partial ^{p}N_{n}(u_{I})}{\partial u_{I}} \biggr\vert \,du_{I} \biggr\Vert &\leq \biggl\Vert \int_{-\delta_{n}}^{\delta_{n}}\cdots \int_{-\delta_{n}}^{\delta_{n}} \biggl\vert \frac{\partial^{p}N_{n}(u_{I})}{\partial u_{I}} \biggr\vert \,du_{I} \biggr\Vert \\ &\leq \int_{-\delta_{n}}^{\delta_{n}}\cdots \int_{-\delta_{n}}^{\delta_{n}} \biggl\Vert \frac {\partial^{p}N_{n}(u_{I})}{\partial u_{I}} \biggr\Vert \,du_{I} \\ &=O\Biggl(\delta_{n}^{q}\sqrt{\sum _{i=1}^{n} \vert x_{in} \vert ^{2+2q}}\Biggr)=O\Biggl(\delta_{n}\sqrt {\sum _{i=1}^{n} \vert x_{in} \vert ^{4}}\Biggr). \end{aligned}$$

Since

4.33 $$ N_{n}(\beta_{n})-N_{n}(0)=\sum _{I\in\{1,2,\ldots,p\}} \int_{0}^{\beta _{n,I}}\frac{\partial^{|I|}N_{n}(u_{I})}{\partial u_{I}}\,du_{I}, $$

the result (4.27) follows from (4.32) and (4.33). □

### Lemma 4.5

*Let* $\pi_{i},i\geq1$ *be a sequence of bounded positive numbers*, *and let there exist a constant* $c_{0}\geq1$ *such that* $\max_{1\leq i\leq2^{d}}\pi_{i}\leq c_{0}\min_{1\leq i\leq2^{d}}\pi _{i}$ *holds for all large* *n*. *And let* $\omega_{d}=2c_{0}\pi_{2^{d}}$ *and* $q>3/2$. *Assume that* (A5) *and* $\tilde{r}_{n}=O(\sqrt{n})$ *hold*. *Then as* $d\rightarrow\infty$

$$\sup_{ \vert \beta_{n} \vert \leq\omega_{d}}\max_{n< 2^{d}} \bigl\vert \tilde{M}_{n}(\beta )-\tilde{M}_{n}(0) \bigr\vert =O_{p} \bigl(\sqrt{\tilde{\tau}_{2^{d}}(\omega_{d})}\,d^{q}+2^{-5d/2} \bigr), $$

*where* $\tilde{M}_{n}(\beta)=\sum_{i=1}^{n}\{\psi(e_{i}-x_{i}^{T}\beta )-E(\psi(e_{i}-x_{i}^{T}\beta)|\mathcal{F}_{i-1})\}x_{i}$.

### Proof

Let

$$\begin{aligned} &\mu_{n}=(\log n)^{q-1},\qquad \tilde{\phi}_{n}=2 \mu_{2^{d}}\sqrt{\tilde{\tau}_{2^{d}}(\omega_{d})}\log \bigl(2^{d}\bigr),\qquad \tilde{t}_{2^{d}}=\mu_{2^{d}}\sqrt{ \tilde{\tau}_{2^{d}}(\omega_{d})}/\log\mu_{2^{d}}, \\ &\tilde{\mu}_{2^{d}}=\tilde{t}_{2^{d}}^{2},\qquad \tilde{\eta}_{i}(\beta)=\bigl(\psi \bigl(e_{i}-x_{i}^{T} \beta\bigr)-\psi(e_{i})\bigr)x_{i},\qquad \tilde{T}_{2^{d}}=\max_{1\leq i\leq 2^{d}}\sup_{ \vert \beta_{n} \vert \leq\omega_{d}} \bigl\vert \tilde{\eta}_{i}(\beta) \bigr\vert \end{aligned}$$

and

$$\tilde{U}_{2^{d}}=\sum_{i=1}^{2^{d}}E \bigl\{ \bigl[\psi\bigl(e_{i}+ \vert x_{i} \vert \omega_{d}\bigr)-\psi \bigl(e_{i}- \vert x_{i} \vert \omega_{d}\bigr)\bigr]^{2}| \mathcal{F}_{i-1}\bigr\} \vert x_{i} \vert ^{2}. $$

Since $q>3/2$ and $2(q-1)>1,\sum_{d=2}^{\infty}(\mu_{2^{d}}^{-1}\log \mu_{2^{d}})^{2}<\infty$. By the argument of Lemma 4.2 and the Borel–Cantelli lemma, we have

4.34 $$ P(\tilde{T}_{2^{d}}\geq\tilde{t}_{2^{d}},i.o.)=0\quad \mbox{and} \quad P(\tilde{U}_{2^{d}}\geq\tilde{u}_{2^{d}},i.o.)=0. $$

Similar to the proof of (4.12), we have

4.35 $$\begin{aligned} &P\Bigl\{ \max_{k\leq2^{d}} \bigl\vert \tilde{M}_{k,j,d}( \beta)- M_{k,j,d}(0) \bigr\vert \geq 2\tilde{\phi}_{2^{d}},\tilde{T}_{2^{d}}\leq\tilde{t}_{2^{d}},\tilde{U}_{2^{d}}\leq \tilde{u}_{2^{d}} \Bigr\} \\ &\quad=O\biggl(\exp\biggl(-\frac{\tilde{\phi}_{2^{d}}^{2}}{4\tilde{t}_{2^{d}}\tilde{\phi}_{2^{d}}+2\tilde{u}_{2^{d}}}\biggr)\biggr). \end{aligned}$$

Let $l=n^{8d}$ and $K_{l}=\{(k_{1}/l,\ldots,k_{p}/l):k_{i}\in Z,|k_{i}|\leq n^{9d}\}$. Then $\#K_{l}=(2n^{9d}+1)^{p}$. By (4.34) and (4.35), for $\forall\varsigma>1$, we have

4.36 $$ P\Bigl\{ \sup_{\beta\in K_{l}} \bigl\vert \tilde{M}_{k,j,d}( \beta)- M_{k,j,d}(0) \bigr\vert \geq 2\tilde{\phi}_{2^{d}},\tilde{T}_{2^{d}}\leq\tilde{t}_{2^{d}},\tilde{U}_{2^{d}}\leq \tilde{u}_{2^{d}} \Bigr\} =O\bigl(n^{-\varsigma dp}\bigr). $$

Therefore,

4.37 $$ P\Bigl\{ \sup_{\beta\in K_{l}} \bigl\vert \tilde{M}_{k,j,d}( \beta)- M_{k,j,d}(0) \bigr\vert \geq 2\tilde{\phi}_{2^{d}},\mathrm{i.o}. \Bigr\} \rightarrow0,\quad n\rightarrow\infty. $$

Since $\tilde{r}_{n}=O(\sqrt{n})$ and $\max_{1\leq i\leq 2^{d}}|x_{i}^{T}(\beta-\langle\beta\rangle_{l,d})|=O(2^{2^{d}}l^{-1}),Cl^{-1}V$ in (4.17) can be replaced by $Cl^{-1}2^{2^{d}}V$, and the lemma follows from $P(V_{2^{d}}\geq2^{5d},\mathrm{i.o}.)=0$. □

### Lemma 4.6

*Let* $\pi_{i},i\geq1$ *be a sequence of bounded positive numbers*, *and let there exist a constant* $c_{0}\geq1$ *such that* $\max_{1\leq i\leq2^{d}}\pi\leq c_{0}\min_{1\leq i\leq2^{d}}\pi _{i}$ *and* $\pi_{n}=o(n^{-1/2}(\log n)^{2})$ *hold for all large* *n*. *And let* $\omega_{d}=2c_{0}\pi_{2^{d}}$. *Assume that* (A6), (A7) *and* $\tilde{r}_{n}=O(\sqrt {n}(\log n)^{-2})$ *hold*. *Then*

4.38 $$ \Bigl\Vert \sup_{ \vert \beta \vert \leq\pi_{n}} \bigl\vert \tilde{N}_{n}( \beta)-\tilde{N}_{n}(0) \bigr\vert \Bigr\Vert =O \Biggl(\sqrt {\sum_{i-1}^{n} \vert x_{i} \vert ^{4}}\pi_{n} \Biggr), $$

*and*, *as* $d\rightarrow\infty$, *for any* $\upsilon>0$,

4.39 $$ \sup_{ \vert \beta \vert \leq\omega_{d}}\max_{n< 2^{d}} \bigl\vert \tilde{N}_{n}(\beta )-\tilde{N}_{n}(0) \bigr\vert ^{2}=o_{a.s.}\Biggl(\sum_{i=0}^{2^{d}} \vert x_{i} \vert ^{4}\omega_{d}^{2}d^{5}( \log d)^{1+\upsilon}\Biggr), $$

*where* $\tilde{N}_{n}(\beta)=\sum_{i=1}^{n}\{\psi_{1}(-x_{i}^{T}\beta ;\mathcal{F}_{i-1})-\varphi(-x_{i}^{T}\beta)\}x_{n}$.

### Proof

Let $Q_{n,j}(\beta)=\sum_{i=1}^{n}\psi_{1}(-x_{i}^{T}\beta ;F_{i-1})x_{ij},i\leq j\leq p$, and

4.40 $$ S_{n}(\beta)=Q_{n,j}(\beta)-Q_{n,j}(0). $$

Note that

4.41 $$ \pi_{n}\tilde{r}_{n}=o\bigl(n^{-1/2}(\log n)^{2}\bigr)O\bigl(\sqrt{n}(\log n)^{-2}\bigr)=o(1). $$

It is easy to see that the argument in the proof of Lemma 4.4 implies that there exists a positive constant $C<\infty$ such that

4.42 $$ E\Bigl\{ \Bigl\vert \sup_{ \vert \beta \vert \leq\omega_{d}} \bigl\vert S_{n}(\beta)-S_{n^{\prime}}(\beta) \bigr\vert \Bigr\vert ^{2}\Bigr\} \leq C\sum_{q=1}^{p} \omega_{d}^{2q}\sum_{i=n^{\prime}+1}^{n} \vert x_{i} \vert ^{2}+2q $$

holds uniformly over $1\leq n'< n\leq2^{d}$. Therefore (4.38) holds.

Let $\Lambda=\sum_{r=0}^{d}\mu_{r}^{-1}$, where

4.43 $$ \mu_{r}=\Biggl\{ \sum_{m=1}^{2^{d-r}} \Bigl\Vert \sup_{ \vert \beta \vert \leq\omega _{d}} \bigl\vert S_{2^{r}m}( \beta)-S_{2^{r}(m-1)}(\beta) \bigr\vert \Bigr\Vert ^{2}\Biggr\} ^{-1/2}. $$

For a positive integer $k\leq2^{d}$, write its dyadic expansion $k=2^{r_{1}}+\cdots+2^{r_{j}}$, where $0\leq r_{j}<\cdots<r_{1}\leq d$, and $k(i)=2^{r_{1}}+\cdots+2^{r_{i}}$. By the Schwartz inequality, we have

4.44 $$\begin{aligned} \sup_{ \vert \beta \vert \leq\omega_{d}} \bigl\vert S_{k}(\beta) \bigr\vert ^{2}&\leq\Biggl\{ \sum_{i=1}^{j} \sup_{ \vert \beta \vert \leq\omega_{d}} \bigl\vert S_{k(i)}(\beta )-S_{k(i-1)}(\beta) \bigr\vert \Biggr\} ^{2} \\ &=\Biggl\{ \sum_{i-1}^{j} \mu_{r_{i}}^{-1/2}\cdot\mu_{r_{i}}^{1/2}\sup _{ \vert \beta \vert \leq\omega_{d}} \bigl\vert S_{k(i)}(\beta)-S_{k(i-1)}( \beta) \bigr\vert \Biggr\} ^{2} \\ &\leq\sum_{i-1}^{j}\mu_{r_{i}}^{-1} \sum_{i-1}^{j}\mu_{r_{i}}\sup _{ \vert \beta \vert \leq\omega_{d}} \bigl\vert S_{k(i)}(\beta)-S_{k(i-1)}( \beta) \bigr\vert ^{2} \\ &\leq\Lambda\sum_{i-1}^{j} \mu_{r_{i}}\sum_{m=1}^{2^{d-\eta }}\sup _{ \vert \beta \vert \leq\omega_{d}} \bigl\vert S_{2^{\eta}m}(\beta)-S_{2^{\eta }(m-1)}( \beta) \bigr\vert ^{2} \\ &\leq\Lambda\sum_{r=0}^{d} \mu_{r}\sum_{m=1}^{2^{d-r}}\sup _{ \vert \beta \vert \leq\omega_{d}} \bigl\vert S_{2^{r}m}(\beta)-S_{2^{r}(m-1)}( \beta) \bigr\vert ^{2}. \end{aligned}$$

Thus

4.45 $$\begin{aligned} \Bigl\Vert \max_{n\leq2^{d}}\sup_{ \vert \beta \vert \leq\omega_{d}} \bigl\vert S_{n}(\beta) \bigr\vert \Bigr\Vert &= \Biggl\Vert \sum _{n=1}^{2^{d}}\sup_{ \vert \beta \vert \leq\omega_{d}} \bigl\vert S_{n}(\beta ) \bigr\vert \Biggr\Vert \\ &\leq\sum_{n=1}^{2^{d}} \Bigl\Vert \sup _{ \vert \beta \vert \leq\omega _{d}} \bigl\vert S_{n}(\beta) \bigr\vert \Bigr\Vert \leq\sum_{n=1}^{2^{d}}\Bigl\{ E \Bigl\vert \sup_{ \vert \beta \vert \leq \omega_{d}} \bigl\vert S_{n}(\beta) \bigr\vert \Bigr\vert ^{2}\Bigr\} ^{1/2} \\ &\leq\sum_{r=0}^{d}\Biggl\{ E \Biggl\vert \Lambda\sum_{r=0}^{d} \mu_{r}\sum_{m=1}^{2^{d-r}} \Bigl\vert \sup_{ \vert \beta \vert \leq\omega_{d}} \bigl\vert S_{2^{r}m}(\beta )-S_{2^{r}(m-1)}(\beta) \bigr\vert \Bigr\vert ^{2} \Biggr\vert \Biggr\} ^{1/2} \\ &\leq\sum_{r=0}^{d}\Biggl\{ \Lambda\sum _{r=0}^{d}\mu_{r}\sum _{m=1}^{2^{d-r}} \Bigl\Vert \sup_{ \vert \beta \vert \leq\omega_{d}} \bigl\vert S_{2^{r}m}(\beta )-S_{2^{r}(m-1)}(\beta) \bigr\vert \Bigr\Vert ^{2}\Biggr\} ^{1/2} \\ &\leq\sum_{r=0}^{d}\Biggl\{ \Lambda\sum _{r=0}^{d} \mu_{r} \mu_{R}^{-2}\Biggr\} ^{1/2}=\sum _{r=0}^{d} \Biggl\{ \Lambda\sum _{r=0}^{d}\mu_{r}^{-1}\Biggr\} ^{1/2}=d\Lambda. \end{aligned}$$

Since $\upsilon>0$ and $\omega_{d}^{2q}\sum_{i=1}^{2^{d}}|x_{i}|^{2+2q}=O(\omega_{d}^{2}\sum_{i=1}^{2^{d}}|x_{i}|^{4})$, (4.42) implies that

4.46 $$ \sum_{d=2}^{\infty}\frac{ \Vert \max_{n\leq2^{d}}\sup_{ \vert \beta \vert \leq\omega_{d}} \vert S_{n}(\beta) \vert \Vert ^{2}}{\omega_{d}^{2}\sum_{i=1}^{2^{d}} \vert x_{i} \vert ^{4}d^{5}(\log d)^{1+\upsilon}} =\sum _{d=2}^{\infty}\frac{O(d^{2}(d+1)^{2})}{d^{5}(\log d)^{1+\upsilon }}< \infty. $$

By the Borel–Cantelli lemma, (4.39) follows from (4.46). □

### Lemma 4.7

*Under the conditions of Theorem *2.2, *we have*:

1. $\sup_{|\beta|\leq b_{n}}|\tilde{K}_{n}(\beta)-\tilde{K}_{n}(0)|=O_{a.s.}(L_{\tilde{n}}+B_{\tilde{n}})$;
2. *for and* $\upsilon>0,\tilde{K}_{n}(0)=O_{a.s.}(h_{n})$, *where* $h_{n}=n^{1/2}(\log n)^{3/2}(\log\log n)^{1/2+\upsilon/4}$.

### Proof

Observe that $\tilde{K}_{n}(\beta)=\tilde{M}_{n}(\beta)+\tilde{N}_{n}(\beta)$. Since $n^{-5/2}=o(B_{\tilde{n}})$, (1) follows from Lemma 4.5 and 4.6. □

As with the argument in (4.29), we have $\tilde{K}_{n}(0)=O(\sqrt{n})$.

### Proof of Theorem 2.1

Observe that

4.47 $$\begin{aligned} K_{n}(\beta_{n})={}&\sum_{i=1}^{n} \psi\bigl(e_{i}-x_{in}^{T}\beta_{n} \bigr)x_{in}-E\Biggl(\sum_{i=1}^{n} \psi\bigl(e_{i}-x_{in}^{T}\beta_{n} \bigr)x_{in}\Biggr) \\ ={}&\sum_{i=1}^{n}\bigl\{ \psi \bigl(e_{i}-x_{in}^{T}\beta_{n}\bigr)-E \bigl(\psi \bigl(e_{i}-x_{in}^{T} \beta_{n}\bigr)|\mathcal{F}_{i-1}\bigr)\bigr\} x_{in} \\ &{}+\sum_{i=1}^{n}\bigl\{ E\bigl(\psi \bigl(e_{i}-x_{in}^{T}\beta_{n}\bigr)| \mathcal {F}_{i-1}\bigr)-E\psi\bigl(e_{i}-x_{in}^{T} \beta_{n}\bigr)\bigr\} x_{in} \\ ={}&M_{n}(\beta_{n})+N_{n}(\beta_{n}). \end{aligned}$$

By (4.47), Lemma 4.2 and Lemma 4.4, we have

4.48 $$\begin{aligned} \sup_{ \vert \beta_{n} \vert \leq\delta_{n}} \bigl\vert K_{n}(\beta_{n})-K_{n}(0) \bigr\vert &\leq\sup_{ \vert \beta_{n} \vert \leq\delta_{n}} \bigl\vert M_{n}( \beta_{n})-M_{n}(0) \bigr\vert +\sup_{ \vert \beta_{n} \vert \leq \delta_{n}} \bigl\vert N_{n}(\beta_{n})-N_{n}(0) \bigr\vert \\ &=O_{p} \bigl(\sqrt{\tau_{n}(\delta_{n})}\log n+n^{-3} \bigr)+O \Biggl(\sqrt{\sum _{i=1}^{n} \vert x_{in} \vert ^{4}}\delta_{n} \Biggr) \\ &=O_{p} \Biggl(\sqrt{\tau_{n}(\delta_{n})}\log n+ \delta_{n}\sqrt{\sum_{i=1}^{n} \vert x_{in} \vert ^{4}} \Biggr). \end{aligned}$$

This completes the proof of Theorem 2.1. □

### Proof of Corollary 2.1

Take an arbitrary sequence $\delta _{n}\rightarrow\infty$, which satisfies the assumption of Theorem 2.1. Note that

4.49 $$ K_{n}(0)=\sum_{i=1}^{n} \psi(e_{i})x_{in}-E\Biggl(\sum _{i=1}^{n}\psi(e_{i})x_{in} \Biggr) =\sum_{i=1}^{n}\psi(e_{i})x_{in} $$

and

4.50 $$\begin{aligned} K_{n}(\hat{\beta}_{n})&=\sum_{i=1}^{n} \psi\bigl(e_{i}-x_{in}^{T}\hat{\beta}_{n}\bigr)x_{in}-E\Biggl(\sum_{i=1}^{n} \psi\bigl(e_{i}-x_{in}^{T}\hat{\beta}_{n} \bigr)x_{in}\Biggr) \\ &=\sum_{i=1}^{n}\psi\bigl(y_{i}-x_{in}^{T} \hat{\beta}_{n}\bigr)x_{in}-\sum_{i=1}^{n} \varphi\bigl(-x_{in}^{T}\hat{\beta}_{n} \bigr)x_{in} \\ &=-\sum_{i=1}^{n}\varphi \bigl(-x_{in}^{T}\hat{\beta}_{n}\bigr)x_{in}+O_{P}(r_{n}) \end{aligned}$$

for $|\hat{\beta}_{n}|\leq\delta_{n}$. By Theorem 2.1 and (4.49), we have

4.51 $$ K_{n}(\hat{\beta}_{n})=\sum_{i=1}^{n} \psi(e_{i})x_{in}+O_{p} \Biggl(\sqrt{\tau _{n}(\delta_{n})}\log n+\delta_{n}\sqrt{ \sum_{i=1}^{n} \vert x_{in} \vert ^{4}} \Biggr). $$

By (4.50) and (4.51), we have

4.52 $$\begin{aligned} &{-}\sum_{i=1}^{n}\varphi \bigl(-x_{in}^{T}\hat{\beta}_{n}\bigr)x_{in}+O_{p}(r_{n}) \\ &\quad= \sum_{i=1}^{n}\psi(e_{i})x_{in}+O_{p} \Biggl(\sqrt{\tau_{n}(\delta_{n})}\log n+\delta_{n} \sqrt{\sum_{i=1}^{n} \vert x_{in} \vert ^{4}} \Biggr). \end{aligned}$$

By (4.52), $\varphi(t)=t\varphi'(0)+O(t^{2})$ as $t\rightarrow0$, and $\sum_{i=1}^{n}x_{in}x_{in}^{T}=I_{p}$, we have

$$\begin{aligned} &{-}\sum_{i=1}^{n}\bigl\{ \bigl(-x_{in}^{T}\hat{\beta}_{n}\bigr)\varphi '(0)+O\bigl(\bigl(-x_{in}^{T}\hat{\beta}_{n}\bigr)^{2}\bigr)\bigr\} x_{in}-\sum _{i=1}^{n}\psi (e_{i})x_{in} \\ &\quad =O_{p} \Biggl(\sqrt{\tau_{n}(\delta_{n})}\log n+ \delta_{n}\sqrt{\sum_{i=1}^{n} \vert x_{in} \vert ^{4}} \Biggr)-O_{p}(r_{n}) \end{aligned}$$

and

$$\begin{aligned} &\sum_{i=1}^{n}x_{in}x_{in}^{T} \varphi'(0)\hat{\beta}_{n}-\sum _{i=1}^{n}\psi(e_{i})x_{in} \\ &\quad =-\sum_{i=1}^{n}O \bigl( \bigl(-x_{in}^{T}\hat{\beta}_{n}\bigr)^{2} \bigr)x_{in}+O_{p} \Biggl(\sqrt{\tau_{n}( \delta_{n})}\log n+\delta_{n}\sqrt{\sum _{i=1}^{n} \vert x_{in} \vert ^{4}} \Biggr)-O_{p}(r_{n}). \end{aligned}$$

Namely

4.53 $$\begin{aligned} &\varphi'(0)\hat{\beta}_{n}-\sum _{i=1}^{n}\psi(e_{i})x_{in} \\ &\quad=O_{p} \bigl(\sqrt{\tau_{n}(\delta_{n})}\log n \bigr) +O_{p} \Biggl(\delta_{n}\sqrt{\sum _{i=1}^{n} \vert x_{in} \vert ^{4}}+\sum_{i=1}^{n} \bigl(-x_{in}^{T}\hat{\beta}_{n}\bigr)^{2}x_{in}+r_{n} \Biggr) \\ &\quad=O_{p} \bigl(\sqrt{\tau_{n}(\delta_{n})}\log n \bigr) +O_{p} \Biggl(\delta_{n}\sqrt{\sum _{i=1}^{n} \vert x_{in} \vert ^{4}}+ \vert \hat{\beta}_{n} \vert ^{2}\sum _{i=1}^{n} \vert x_{in} \vert ^{3}+r_{n} \Biggr) \\ &\quad=O_{p} \bigl(\sqrt{\tau_{n}(\delta_{n})}\log n \bigr)+O_{p} \bigl(\delta _{n}r_{n}+ \delta_{n}^{2}r_{n}+r_{n} \bigr) \\ &\quad=O_{p} \bigl(\sqrt{\tau_{n}(\delta_{n})}\log n+ \delta_{n}^{2}r_{n} \bigr). \end{aligned}$$

By $m(t)=O(|t|^{\lambda})\ (t\rightarrow0)$ for some $\lambda>0$, we have

4.54 $$ \tau_{n}(\delta_{n})=2\sum_{i=1}^{n} \vert x_{in} \vert ^{2}\bigl( \vert x_{in} \vert \delta _{n}\bigr)^{2\lambda}=2\delta_{n}^{2\lambda} \sum_{i=1}^{n} \vert x_{in} \vert ^{2+2\lambda}. $$

Then it follows from (4.53) and (4.54) that

4.55 $$\begin{aligned} &\varphi'(0)\hat{\beta}_{n}-\sum _{i=1}^{n}\psi(e_{i})x_{in} \\ &\quad=O_{p} \bigl(\sqrt{\tau_{n}(\delta_{n})}\log n+\delta_{n}^{2}r_{n} \bigr) \\ &\quad=O_{p} \Biggl(\sqrt{\sum_{i=1}^{n} \vert x_{in} \vert ^{2+2\lambda}}\delta _{n}^{\lambda}\log n+\delta_{n}^{2}r_{n} \Biggr) \end{aligned}$$

for any $\delta_{n}\rightarrow\infty$, which implies

4.56 $$ \varphi'(0)\hat{\beta}_{n}-\sum _{i=1}^{n}\psi(e_{i})x_{in}=O_{p} \Biggl(\sqrt {\sum_{i=1}^{n} \vert x_{in} \vert ^{2+2\lambda}}\log n+r_{n} \Biggr). $$

 □

### Proof of Theorem 2.2

By Lemma 4.7, we have Theorem 2.2. □

### Proof of Corollary 2.2

(1) By Lemma 4.7, we have

4.57 $$ \sup_{ \vert \beta_{n} \vert \leq b_{n}} \bigl\vert \tilde{K}_{n}( \beta_{n}) \bigr\vert \leq\sup_{ \vert \beta_{n} \vert \leq b_{n}} \bigl\vert \tilde{K}_{n}(\beta_{n})-\tilde{K}_{n}(0) \bigr\vert +\tilde{K}_{n}(0)=O_{a.s.}(L_{\tilde{n}}+B_{\tilde{n}}+h_{n}), $$

where $b_{n}=n^{-1/2}(\log n)^{3/2}(\log\log n)^{1/2+\upsilon}$. Let

4.58 $$ \Theta_{n}(\beta)=\sum_{i=1}^{n} \bigl[\rho\bigl(e_{i}-x_{i}^{T}\beta\bigr)- \rho(e_{i})\bigr] $$

and

4.59 $$ A_{n}(\beta)=-\sum_{i=1}^{n} \int_{0}^{1}\varphi\bigl(-x_{i}^{T} \beta\bigr)x_{i}^{T}\beta \,dt. $$

Note that

4.60 $$ \rho(e_{i})-\rho\bigl(e_{i}-x_{i}^{T} \beta\bigr)= \int_{0}^{1}\psi\bigl(e_{i}-x_{i}^{T} \beta\bigr)x_{i}^{T}\beta \,dt. $$

By (4.57)–(4.60), we have

4.61 $$\begin{aligned} \sup_{ \vert \beta_{n} \vert \leq b_{n}} \bigl\vert \Theta_{n}( \beta)-A_{n}(\beta) \bigr\vert &=\sup_{ \vert \beta_{n} \vert \leq b_{n}} \Biggl\vert \sum_{i=1}^{n} \int_{0}^{1}\bigl[\psi\bigl(e_{i}-x_{i}^{T} \beta \bigr)-\varphi\bigl(-x_{i}^{T}\beta\bigr) \bigr]x_{i}^{T}\beta \,dt \Biggr\vert \\ &=\sup_{ \vert \beta_{n} \vert \leq b_{n}} \biggl\vert \int_{0}^{1}\tilde{K}_{n}(\beta t)\beta \,dt \biggr\vert \\ &=O_{a.s.}\bigl((L_{\tilde{n}}+B_{\tilde{n}}+h_{n})b_{n} \bigr). \end{aligned}$$

It is easy to show that $b_{n}^{3}\sum_{i=1}^{n}|x_{i}|^{3}=O(n\tilde{r}_{n})b_{n}^{3}=o(nb_{n}^{2})$. By $\varphi(t)=t\varphi'(0)+O(t^{2})$, we have

4.62 $$\begin{aligned} \inf_{ \vert \beta_{n} \vert = b_{n}}A_{n}(\beta)&=\inf_{ \vert \beta_{n} \vert = b_{n}} \Biggl\{ -\sum_{i=1}^{n} \int_{0}^{1}\varphi\bigl(-x_{i}^{T} \beta\bigr)x_{i}^{T}\beta \,dt\Biggr\} \\ &=\inf_{ \vert \beta_{n} \vert = b_{n}}\Biggl\{ -\sum_{i=1}^{n} \int_{0}^{1}\bigl[\varphi (0)+\varphi'(0) \bigl(-x_{i}^{T}\beta\bigr)+O\bigl(\bigl(-x_{i}^{T} \beta\bigr)^{2}\bigr)\bigr]x_{i}^{T}\beta \,dt\Biggr\} \\ &=\inf_{ \vert \beta_{n} \vert = b_{n}}\Biggl\{ \sum_{i=1}^{n} \biggl[\frac{1}{2}\varphi '(0) \bigl(-x_{i}^{T} \beta\bigr)^{2}|_{0}^{1}-\frac{1}{3}O\bigl( \bigl(-x_{i}^{T}\beta\bigr)^{3} \bigr)|_{0}^{1}\biggr]\Biggr\} \\ &=\frac{1}{2}\varphi'(0)\sum_{i=1}^{n} x_{i}^{T} x_{i}b_{n}^{2}- \frac {1}{3}\inf_{ \vert \beta_{n} \vert = b_{n}}\Biggl\{ b_{n}^{2} \sum_{i=1}^{n}O\bigl(x_{i}^{T} x_{i}\bigr)x_{i}^{T} \beta\Biggr\} \\ &\geq\frac{1}{2}\varphi'(0)S_{n}b_{n}^{2}- \frac{1}{3}b_{n}^{2}\sum_{i=1}^{n} \vert x_{i} \vert ^{3}b_{n}O(1) \\ &\geq\frac{1}{6}\varphi'(0)nb_{n}^{2} \liminf_{n\rightarrow\infty }\lambda_{n}/n. \end{aligned}$$

By $m(t)=O(\sqrt{n})$ as $t\rightarrow0$, we have $(L_{\tilde{n}}+B_{\tilde{n}}+h_{n})b_{n}=o(nb_{n}^{2})$. Thus

4.63 $$\begin{aligned} \inf_{ \vert \beta_{n} \vert = b_{n}}\Theta_{n}(\beta)&\geq\inf _{ \vert \beta_{n} \vert = b_{n}}A_{n}(\beta)-\sup_{ \vert \beta_{n} \vert \leq b_{n}} \bigl\vert \Theta_{n}(\beta)-A_{n}(\beta ) \bigr\vert \\ &\geq\frac{1}{6}\varphi'(0)nb_{n}^{2} \liminf_{n\rightarrow\infty }\lambda_{n}/n+O_{a.s.} \bigl((L_{\tilde{n}}+B_{\tilde{n}}+h_{n})b_{n}\bigr) \\ &\geq\frac{1}{4}\varphi'(0)nb_{n}^{2} \liminf_{n\rightarrow\infty} \lambda_{n}/n,\quad \mbox{a.s.} \end{aligned}$$

By the convexity of the function $\Theta_{n}(\cdot)$, we have

4.64 $$\begin{aligned} &\biggl\{ \inf_{|\beta_{n}|\geq b_{n}}\Theta_{n}(\beta)\geq \frac{1}{4}\varphi '(0)nb_{n}^{2}\liminf _{n\rightarrow\infty}\lambda_{n}/n\biggr\} \\ &\quad =\biggl\{ \inf_{|\beta_{n}|= b_{n}}\Theta_{n}(\beta)\geq \frac{1}{4}\varphi '(0)nb_{n}^{2}\liminf _{n\rightarrow\infty}\lambda_{n}/n\biggr\} . \end{aligned}$$

Therefore the minimizer $\hat{\beta}_{n}$ satisfies $\hat{\beta}_{n}=O_{a.s.}(b_{n})$.

(2) Let $|\hat{\beta}_{n}|\leq b_{n}$. By a Taylor expansion, we have

4.65 $$\begin{aligned} -\sum_{i=1}^{n}\varphi \bigl(-x_{i}^{T}\beta\bigr)x_{i}&=\sum _{i=1}^{n}\bigl[\varphi '(0)x_{i}^{T} \beta+O\bigl( \bigl\vert x_{i}^{T}\beta \bigr\vert ^{2}\bigr)\bigr]x_{i} \\ &=\varphi'(0)\Sigma_{n}\beta+O\Biggl(b_{n}^{2} \sum_{i=1}^{n} \vert x_{i} \vert ^{3}\Biggr). \end{aligned}$$

Therefore (2) follows from Theorem 2.2 and (1). □
